# Development of A Tool for Assessing the Reputation of Zoos: The Zoo Ethical Reputation Survey (ZERS)

**DOI:** 10.3390/ani12202802

**Published:** 2022-10-17

**Authors:** Maria Michela Spiriti, Francesco Maria Melchiori, Paul Wilhelm Dierkes, Linda Ferrante, Francesca Bandoli, Pierfrancesco Biasetti, Barbara de Mori

**Affiliations:** 1 Department of Comparative Biomedicine and Food Science, University of Padua, 35020 Padua, Italy; 2 Ethics Laboratory for Veterinary Medicine, Conservation and Animal Welfare, University of Padua, 35020 Padua, Italy; 3 Faculty of Psychology, University Niccolò Cusano, 00166 Roma, Italy; 4 Department for Bioscience Education, Goethe University, 60438 Frankfurt, Germany; 5 Giardino Zoologico di Pistoia, 51100 Pistoia, Italy; 6 Department of Reproduction Management, Leibniz Institute for Zoo and Wildlife Research, 10315 Berlin, Germany

**Keywords:** ethical reputation, zoo, zoo corporate reputation, biodiversity conservation, zoo management, ethical tools

## Abstract

**Simple Summary:**

The reputation of a zoo indicates the level of public consideration of this institution and is determined by the actions, values, and behaviors that it has conveyed over time. The reputation of zoos is a complex construct and highlighting the key factors that can negatively affect it can lead to identifying ways to promote their reputation. To address these critical issues, a zoo must not only promote higher operational and ethical standards and animal welfare but also be certain that the stakeholders perceive the importance of its mission. This will benefit the individual institution and zoological institutions as a whole as a positive reputation will enable zoos to thrive in the future as biodiversity conservation institutions and places of environmental education and entertainment publicly supported. In this work, we report the development and the first trial of the Zoo Ethical Reputation Survey (ZERS), a tool that, through a survey designed with ad hoc items, analyzes public opinion on features that can influence the reputation of a zoo, focusing on ethical aspects. During its first applications, ZERS proved to be a tool able to provide information on the visitors’ opinions about several drivers that, according to the literature, influence corporate reputation.

**Abstract:**

Nowadays, most zoos have taken prominent and active positions in endangered species conservation and educating visitors about the value of biodiversity. However, to be effective and trusted in their mission, they must act ethically and have a good reputation. Yet, the drivers that can influence their reputation are still little investigated, and there are still few studies focused on assessing the reputation of these institutions. In the present work, we report the development of a tool, the Zoo Ethical Reputation Survey (ZERS), and its pilot application to assess the opinions of the visitors of two zoos, one in Italy and one in Germany, on drivers that may influence the ethical reputation of zoos. Preliminary results based on the answers of 274 respondents show that visitors’ opinions on zoos acting with ethical responsibility are correlated with emotional appeal and familiarity with these institutions. The application of ZERS can help zoos identify weaknesses in their reputation and develop new strategies to improve people’s attitudes towards them, bringing many benefits to the individual zoo and zoological institutions in general.

## 1. Background

More than 700 million people, one-tenth of the world population, representing a wide variety of demographic categories, visit zoos every year [1,2,3,4]. With such vast and wide-ranging audiences, zoos can play an important role in educating children and adults on the importance of biodiversity and raising awareness of conservation challenges [2]. Zoos are facilitated in their role by the fact that, while providing an entertainment experience, they create in visitors an emotional connection with animals and their stories [5,6]. Moreover, the zoo experience itself provides visitors with implicit emotional connections with Nature as these institutions represent the first—and often the only—place where people can encounter many different species of wild animals [3]. These emotional connections are important because they have been seen to generate a motivational stimulus that eases the learning of ethological and ecological contents, making visitors more receptive to conservation messages [3,7,8,9,10,11].

Over the years, zoos have progressively assumed active and prominent positions in wildlife research and biodiversity conservation, supporting an integrated approach to species protection, like the One Plan Approach [12,13]. This conservation strategy—in which zoos play a relevant role—helps to bridge the gap between wild and captive population management, involving all conservationists (e.g., field biologists, wildlife managers, zookeepers, etc.) to develop a shared planning tool useful for species conservation [13,14]. However, to fulfill their mission, zoos must be trustworthy and credible in their role. For this reason, they need to have a good reputation among the public and other stakeholders.

The concept of the reputation of a zoo can be regarded as the application to zoological institutions of the well-known marketing concept of corporate reputation. According to Fombrun and Van Riel, corporate reputation is a collective representation of a firm’s past actions and results that describes the firm’s ability to deliver valued outcomes to multiple stakeholders [15]. Similarly, the reputation of a zoo can be defined as the collective representation of its past actions, commitment, and ability to fulfill its mission. It represents the general esteem in which the zoo is held internally by employees and externally by its stakeholders.

**Figure 1:**
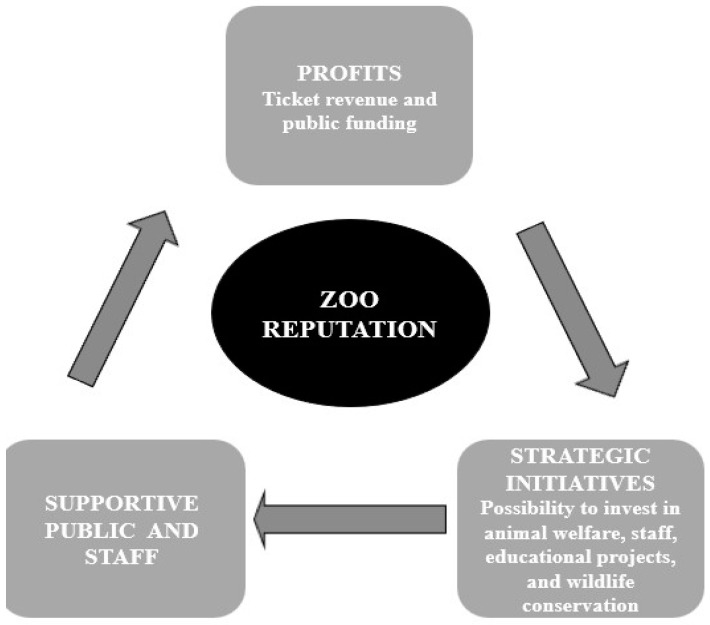
The reputational value cycle of zoos. A good zoo reputation can act as a positive reinforcement loop engine. It will ensure supportive public and staff, attract more visitors and revenue, and provide access to public funding. These will allow investment in strategic initiatives (animal welfare, staff, educational projects, and wildlife conservation), enabling the zoo to act according to its mission.

Reputation is considered an intangible but highly valuable asset. Indeed, studies have shown that corporate reputation has surpassed traditional palpable assets in determining the ability of a company to thrive because it attracts public support and more and better resources [16,17]. Likewise, also for zoological institutions, a positive reputation can produce several benefits. For instance, zoos with a positive reputation can attract more visitors, build loyalty, gain their trust and support for their conservation projects, be more effective in their pro-conservation messages, and have easier access to funds. As a result, a positive reputation can fuel a positive “reinforcement loop” that facilitates the fulfillment of their institutional mission (Figure 1).

Furthermore, the benefits of a positive reputation reflect not only on the individual zoo but also on the whole zoo community. It may lead to a virtuous cycle in other zoos, encouraging them to operate at the highest standards and act ethically. Above all, the ethical aspects involved in the activities of zoos are becoming progressively crucial in contributing to a good reputation of these institutions as ‘ethical arks’ [18]. These aspects can be listed as, for example, acting responsibly towards their mission, promoting individual animal welfare while enhancing the chance for conservation of species, promoting transparency within the public in educational efforts, and selecting to adhere to conservation projects based on common ethical standards [4,14,19,20,21,22]. Zoological associations can benefit from analyzing and monitoring the reputation of their members and setting high ethical and reputational standards to which they must adhere. 

Only zoos with a good reputation are considered credible in their actions as institutions for biodiversity protection and education by visitors, the general public, and the social networks in which they operate. Hence, there is an increasing need for zoological associations and individual zoos to be able to identify the crucial aspects that may influence their reputation. To our knowledge, currently, there are no existing tools able to evaluate the reputation—and specifically the ethical reputation—of zoos among visitors. Therefore, we designed an ad hoc survey, the Zoo Ethical Reputation Survey (ZERS). Here, we present its development and the results of its first trial in two zoos, one in Italy and one in Germany.

## 2. Method

### 2.1. The Conceptual Framework of ZERS

The first step in the design of ZERS consisted of a literature review on corporate reputation. The literature on the topic was retrieved from Scopus and Google scholar using the Boolean strings of the following combination of keywords (“corporate” or “zoo” or “zoos” “zoological garden” or “zoological gardens”) AND “reputation”. The retrieved articles were analyzed to identify the reputational key drivers, that is, the factors that drive corporate reputation by influencing and shaping it. Subsequently, the literature on each identified key driver was further investigated, and the concepts found were adapted to the context of zoological institutions.

There are many theoretical frameworks concerning possible drivers for reputation, with no consensus on their real action and effectiveness. The difficulty in identifying which drivers influence reputation unambiguously is partly due to the fact that a universal and operational definition of reputation is lacking because the concept needs to be defined each time for different contexts [15,17,23]. This is particularly evident in zoos, which are very complex entities dealing with multiple stakeholders with very different and sometimes contradictory interests (e.g., individual animals, visitors, wildlife species, social communities, etc.). Consequently, many, often interconnected, factors can affect the reputation of zoos among the public. 

**Figure 2:**
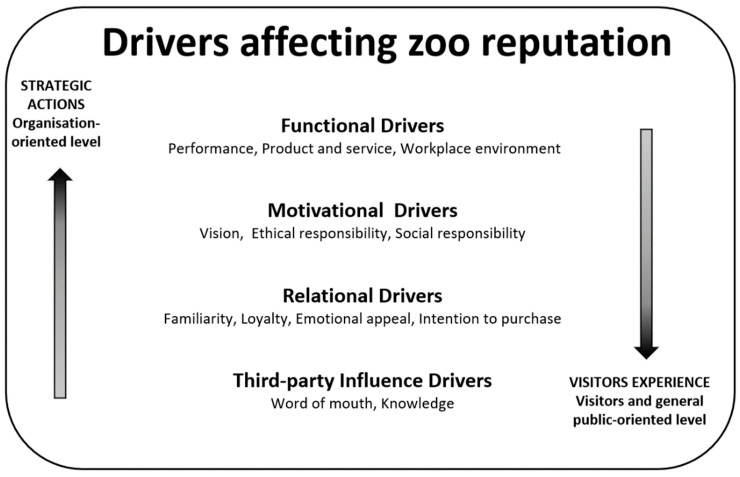
Drivers affecting zoo reputation analyzed in ZERS.

For the development of ZERS, four types of drivers that may affect visitor opinions were considered: functional drivers, motivational drivers, relational drivers, and third-party influence drivers (Figure 2). Moreover, particular attention was paid to the ethical aspects concerning the activities of zoos. Analyzing and addressing the most pressing ethical issues concerning zoos is crucial not only to give deeper meaning to the maintenance of wildlife in these facilities but, above all, not to provide ammunition to those who oppose the very existence of zoos [4].

#### 2.1.1. Functional Drivers

Functional drivers are related to the running of zoos and are the most widely researched in zoo management. They are affected by visitors’ experiences of products, services, performance, and the working environment of the zoo, and they give the perceptions of the quality, innovation, value, and reliability of the institution’s products and services [24]. The performance represents the potential and ability of an organization to efficiently utilize the available resources to achieve targets in line with the set plans, keeping in mind their relevance to the stakeholders [25].

For a zoo, this means achieving the goals of its mission taking into consideration visitor satisfaction. The performance evaluation of a zoo is very important for investigating the quality of animal exhibits, husbandry and care of the animals, educational programs, and conservation projects. The analysis of the performance can help zoos maximize their education and conservation activities, encouraging them to work at higher standards and identify particular issues or concerns [26,27]. In addition, setting performance benchmarks can also help improve individual institutions and the zoological industry as a whole [28]. Moreover, the performance of a zoo is connected to the employees’ working conditions and satisfaction. Specifically, good working conditions promote a connection between the employees, the zoo, and its mission. Subsequently, there will be less turnover, and the higher level of skills and know-how of employees will positively impact the performance of the zoo. Furthermore, the public will be more likely to believe that the institution and its workers are credible and dedicated to their mission [29]. 

Zoos are also places of entertainment, and customers who visit them expect to have a pleasant time there. Therefore, a positive experience of the performance, products, and services of the zoo during the visit significantly influences visitors’ satisfaction, their intent to revisit, and their opinion about the reputation of the zoo [30,31].

#### 2.1.2. Motivational Drivers

Motivational drivers are related to the vision of the zoo and its social and ethical responsibility. Vision integrates the mission, the purpose of the organization, and values into a cohesive action-oriented plan [32]. Especially, the mission of the zoo should be clearly expressed and declined in action-oriented language so that their accomplishment can also be evaluated by the general public [33]. The adherence of the zoo to its stated vision and the achievement of its goals can significantly influence public opinion and, consequently, the reputation of the zoo. Furthermore, zoos should cultivate a relationship with visitors to encourage them to identify with their mission to entice them to participate in their conservation efforts. However, the good reputation of a zoo is also established by the social role it can play and its ethical responsibility. In particular, its commitment to social and ethical responsibility is crucial. Zoo social responsibility is the ability to promote projects involving local communities and be an environmentally responsible organization. A corporation that acts according to socially responsible principles and practices is perceived as a good citizen in its dealings with the community, employees, and the environment, and its reputation will undoubtedly benefit from this [16,34]. Similarly, also the ethical responsibility of a zoo significantly impacts its reputation. Acting according to ethical responsibility leads zoos to operate transparently, be open and accurate when disseminating information, and be committed to advancing superior animal welfare standards and practices [18,20].

#### 2.1.3. Relational Drivers

The relational drivers that can influence the reputation of a zoo are related to the relationship with its visitors, such as its emotional appeal among the public and the familiarity and loyalty of its visitors, as well as visitors’ repurchasing intentions. Zoos should create an emotional bond with their visitors so that communication of the pro-conservation messages can reach not only their minds but also their hearts [35]. This emotional bond motivates visitors towards a personal commitment to Nature through donations to support projects carried out by zoos, as has been observed for other organizations [36]. More importantly, this affective component generates a place attachment. This loyalty to a particular zoo can be easily translated into a familiarity with zoological institutions in general, which increases esteem in these organizations and the likelihood of revisiting or visiting other zoos in the future and even recommending them to others [37,38,39]. Any zoo should succeed in creating this attachment in its visitors because this will facilitate the achievement of its mission. Indeed, research suggests that repeat visitors are more likely to seek conservation efforts than those visiting zoos for the first time [40,41,42].

#### 2.1.4. Drivers of Third-Party Influence

Third-party drivers that can influence the reputation of a zoo are related to the multi-way communication between the zoo and visitors, the general public, zoo networks, etc. Therefore, a zoo must know what kind of information is provided about it and how it is spread. Especially the dissemination of information through direct word of mouth among acquaintances significantly impacts reputation, as opinions conveyed in this way are often considered more trustworthy than those reported by other sources [43,44]. Recently, this way of disseminating information has become even more relevant in shaping reputation because, through the Internet, electronic word of mouth (eWOM) can be spread globally, even among people who have never met each other, with a greater effect. Moreover, the more people publicly share that opinion, the bigger will be the number of people who agree with it. This is caused by a psychological phenomenon known as the “bandwagon effect”, which generates a mechanism of social self-reinforcing in which the spreading of an opinion by the majority induces individuals to adopt that opinion as their own regardless of its veracity [45].

### 2.2. ZERS

**Figure 3:**
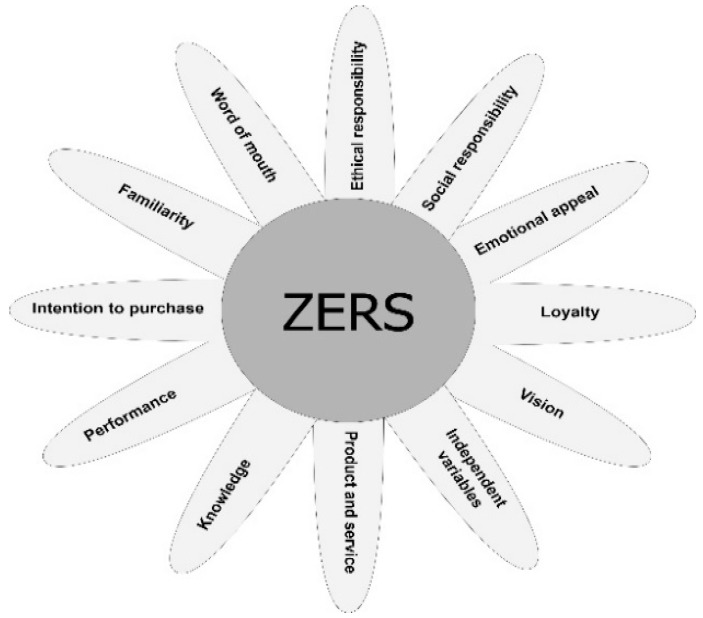
ZERS outline shows the drivers influencing the reputation of a zoo analyzed in the tool.

As previously described, the review of the corporate reputation literature allowed us to select the categories of drivers that could be used in the analysis of the reputation of zoos. These drivers were utilized to define the ZERS outline (Figure 3), and, for each driver, the most critical issues that can influence the reputation of a zoo were highlighted and analyzed.

Consequently, we inserted 53 items in the ZERS survey to reflect these facets and were used to measure the opinion of visitors with the aim of implementing relevant strategies to address them. Furthermore, 9 additional questions were inserted to record their demographic characteristics. A challenging questionnaire in length for respondents but similar in length to questionnaires created to investigate the corporate reputation of other institutions [46]. We applied a psychometric methodology to formulate different kinds of items (i.e., closed-ended multiple-choice questions, rating scale questions, and Likert scale questions) depending on the type of information to be collected by the interviewees [47]. In the survey, the 5-point Likert scale items assessed the visitors’ attitudes (options ranging from Strongly Disagree, Disagree, Neither Agree nor Disagree, and Strongly Agree). While we used a rating scale ranging from 1 (not at all likely) to 5 (extremely likely) to measure opinions such as the likelihood that visitors would recommend zoological institutions or visit a zoo in the future.

In the questionnaire, the items were not subdivided or ordered according to the different categories shown in Figure 2 but according to the order considered easiest for respondents to answer. In any case, they were placed in such a way that respondents could not figure out to which reputational drivers they were referring, to avoid response bias. Table 1 shows some of the questionnaire items for each specific facet.

### 2.3. The Administration of ZERS 

The first trial of ZERS was in a two-site cross-sectional observational study, a method used to compare the opinions of two different groups of zoo visitors at one point in time [48]. Specifically, ZERS was administered to visitors in two European zoos: the Zoological Gardens of Pistoia in Italy and the Opel Zoo in Germany. The researchers administered the survey to visitors following a random sampling procedure and fairly sampled visitors that passed an imaginary line in front of them [48,49].

All the participants were informed of the purpose of the research, and verbal consent was requested when they were invited to take part in the study. Permission from responsible adults was sought before potential respondents of minor age were approached. No anticipated risks to the participants were identified as they were invited to take part voluntarily and anonymously in the study at the entrance of the zoo. Furthermore, to ensure anonymity, no personal data that could link the questionnaire to the respondent’s identity in any possible way were collected. The administration of the questionnaire took place in both the zoological institutions, for approximately seven hours per day, on 2nd and 3rd June 2018, from 10 a.m. until closing time.

### 2.4. Methods and Reliability Analysis

The research hypothesis had a twofold focus: to analyze how visitors in the two different zoos perceive the reputation and ethical aspects of the activities of the zoos and to investigate which drivers influence them. 

Propaedeutically to the data analysis, a study of ZERS questionnaire reliability was performed to identify which dimensions to retain. R. and Jamovi software were used for all analysis and plots [50,51,52]. For this purpose, Cronbach’s coefficient α was used to calculate the internal consistency coefficients of the scales. This coefficient represents how closely related a set of items are as a group, that is, how stable measurement is, as it is a requirement for validity.

As shown in Table 2, the 95% confidence intervals of Cronbach’s α for all the drivers/dimensions include a parameter of around 0.70 (except in the case of Loyalty driver). Given the early stage of this construct validation research, such reliability value was considered satisfactory, although modest for Nunnally and Bernstein standards [53].

As previously stated, reliability is a necessary condition for validity, but it does not imply it. Although the numerosity of respondents did not provide the opportunity for a more advanced statistical analysis of the ZERS validity, the correlation among key drivers was used to test our hypothetical pattern. Based on the theoretical development of the ZERS tool, if the drivers were valid in the measurement, we expected a stronger relationship between all other variables, as theoretically hypothesized. In fact, the correlation matrix (Table 3) provided indications of a statistically significant moderate positive correlation between ethical responsibility (ETR) and emotional appeal (EMA), r(263) = 0.581, *p* < 0.01, indicating how the perception of zoo mission can also activate emotional arousal in the visitors (and vice versa). Similarly, the small positive correlation between familiarity zoo-related (FAM_ZOO), r(239) = 0.133, *p* < 0.05 and familiarity with other settings such as parks and aquariums (FAM_NO-ZOO), r(235) = 0.335, *p* < 0.01 was expected because it intercepts the profile of people who like visiting natural attractions. All the other correlations between the selected key drivers are smaller but statistically significant, confirming that they represent different but related dimensions of the zoo reputation construct.

This evidence was considered to support the data analysis related to the questionnaire dimensions, except for the Loyalty driver, which was considered biased and was not taken into further consideration.

## 3. Results

Three hundred thirty-three respondents filled out the questionnaire. After the data screening (checking for missing data, uncompleted or unengaged responses, etc.), the final dataset analyzed comprised 274 data points: 89 (32.8%) in Germany and 189 (67.2%) in Italy. This step of data analysis can also be considered a preliminary phase, as it regards the comparison of the two populations to highlight relevant differences. This comparison can provide additional insight into the discussion of the results related to the ZERS drivers.

To investigate the socio-demographic characteristics of the visitors surveyed, respondents of the two different zoos were compared on the main variables using the chi-square test of independence. The two groups demonstrated statistically significant differences in gender (χ^2^ = 24.45, *p* < 0.001), with 52.2% male respondents in the Italian zoo and 31.7% in the German zoo. This difference in gender proportions in the two populations highlighted by the Chi square statistics is relevant because literature reports gender differences in customer expectations and perceptions of corporate social responsibility in other contexts [54]. Moreover, visitors of the Italian zoo had a statistically significantly higher age (rrb = −0.63, *p* < 0.01), with a median age of 35–54 years, while the median age of visitors of the German zoo was 26–34 years. Rank-biserial correlation value between one nominal variable (nationality) and one continuous one (age) is important because age can affect some reputation drivers, as shown by our results, a little further. Therefore this may explain the higher mean scores of the items. Moreover, the education level of the visitors to the Italian zoo was significantly higher (rrb = −0.21, *p* < 0.01), with 82.3% of Italian visitors having a secondary school diploma or a higher education compared to 66.9% of the visitors of German zoo, but a lower income (rrb = −0.385, *p* < 0.01), with Italians having income level median of 14,000−29,999 € and Germans of 30,000−40,000 €. Education and income levels did not appear relevant for reputation drivers in the following analysis. Therefore, these differences could be negligible.

A descriptive analysis of the responses to single items was also conducted to better comprehend the participants’ perception, and to test the usefulness of ZERS tool in this trial. Additionally, supplementary evaluations on the responses in the two zoos were conducted on some ad-hoc selected items using the Mann–Whitney U test, because the variables were considered as ordinal in nature. For all these items, the mean value of the Italian population was higher than the German one; in fact, the W scores are positive, but only a few of these differences are statistically significant (Table 4). For example, question 21, reflecting performance driver (*p* < 0.001), shows how Italian respondents perceive that “Zoos dedicate themselves to conservation projects in the wild” more than the German group. This information could be used, for example, as leverage in media campaigns, etc. 

More results are described in Table 2, and descriptive plots and further descriptive analysis are reported in the supplementary materials (S2).

Next, the different effects between nationalities on the relevant drivers (continuous variables) regarding visitors’ opinions on familiarity (FAM), ethical responsibility (ETR), and emotional appeal (EMA) were checked with gender as a grouping variable, using the Independent Samples T-Test (Table 5).

Regarding visitors’ opinions, the differences between nationality on the relevant drivers (continuous variables) on familiarity (FAM), ethical responsibility (ETR), and emotional appeal (EMA) were checked with gender as a grouping variable, using the Independent Samples T-Test. The objective, in this case, was to verify a possible effect of gender within the nationality. The results presented in Table 5 confirm for all the drivers (except for familiarity) a higher statistically significant perception for male visitors versus female ones (positive mean difference and *p*-value < 0.05). Zoos could evaluate this evidence to reflect on the reasons why there is this difference and how to intervene to raise the perception of female visitors.

**Figure 4:**
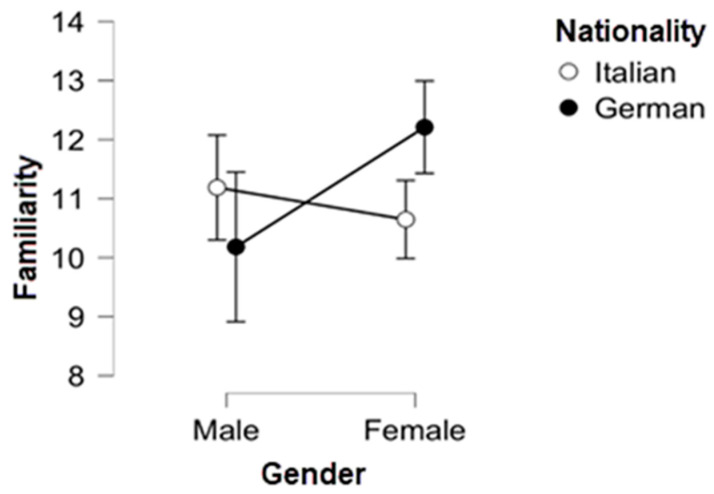
Descriptive plots of the statistically significant interaction GENDER × NATIONALITY on familiarity. Axes: Y = Familiarity scores; X = gender. The descriptive plots of the other items can be found in supplementary materials S2.

Further analysis with two-way ANOVA highlighted differences in familiarity (FAM) considering the nationality and gender variables at the same time. The statistic coefficients showed that while the principal effects of the independent variables (the “gender” and “nationality” rows) are not statistically significant, their interaction (the “GENDER × NATIONALITY” row) is determining an effect (*p* = 0.027) on the dependent variable “Familiarity” (Table 6 and Figure 4). This result explains the opposite trend presented in Table 5 because the plot in Figure 4 shows a statistically significant difference of familiarity mean scores between German female visitors and Italian ones. This test value may be due to the unbalanced gender distribution in the German sample. Still, it may be worth investigating in the future because this opposite trend can be determined by other intervening variables (like a ticket price policy favourable to female visitors that increase their familiarity with these structures).

Complementarily, Post Hoc Tests were conducted to evaluate the differences between the combination of gender and nationality of the respondents in the two zoos to complete the model description, as reported in Table 7. This results are more interesting when considering that independent T-test on familiarity examining only nationality shows an higher mean for German sample t(215) = -2.090, p = 0.038.

Finally, data of all respondents were analyzed as a whole, and two multiple regressions were run to predict differences in emotional appeal (EMA) and ethical responsibility (ETR) from gender, age, and education level (EDL), pet ownership (PETOWN), urbanization (URBANIZ), income level (INCOME), and zoo familiarity (FAM-ZOO). Both multiple regression model statistically significantly predicted the dependent variables (EMA: F(7, 218) = 2.267, *p* = 0.03, adj. R2 = 0.038; ETR: F(7, 215) = 2.842, *p* = 0.007, adj. R^2^ = 0.056) with small effect sizes according to Cohen’s classification [55]. In both models, age and zoo familiarity were found to be significant predictors (*p* < 0.05), and this consistency may indicate these are two variables affecting the reputation construct as a whole. Regression coefficients and standard errors showed how an increase in zoo familiarity and age determines a rise in emotional appeal and ethical responsibility, as presented in Table 8 and Table 9. The positive sign of the β coefficients indicates that older people with a better familiarity with the zoo also perceive more emotional appeal toward it and its ethical responsibility and vice versa.

## 4. Discussion

The results of the preliminary analysis regarding the validity and reliability of the ZERS revealed a positive evaluation of both internal consistency and construct validity. This analysis confirmed the quality of the tool regarding eight scales/drivers and suggested complete revision of the Loyalty scale, which presented an inadequate Cronbach’s α value and, consequently, a low level of construct validity. Additional technical issues are reported in the limitation section. However, further testing is required to validate the instrument, collecting more numerous samples to implement more advanced psychometric methods and, ultimately, developing a quotient that can quantitatively measure the reputation of zoos among the public, as has been done for other corporations [24].

Furthermore, the responses to the questionnaires were analyzed for preliminary socio-demographic information of the respondents in the two countries presented some interesting differences. The results showed that the Italian respondents were mainly men and had statistically higher age, education level, and income. This is probably due to the fact that families with children often visit zoos, and Italians tend to have children later in life when they have completed their studies [56]. In otheYes it ensure the originals meaning r studies, it was observed that educational background and income level influence the extent to which people visit zoos [57,58]. In fact, even if humans seem to be characterized by an innate “biophilic instinct” [59], research shows that a higher level of education is correlated with greater interest and affection for Nature [60]. Presented results do not support this claim regarding the zoo reputation construct although familiarity appears to influence its key drivers like Emotional Appeal and Ethical Responsibility. Nevertheless, apart from age, no other socio-demographic variable appears to influence key drivers. One explanation is that reputation of a zoo is a multi-facet construct that requires a long time to be acquired. Zoos and other stakeholders can use this information to calibrate their communication, e.g., it would be inefficient to focus on children to improve this construct. 

Moreover, the differences in perceptions of the key drivers of the reputation of the zoo between male visitors and female ones were consistent across all the dimensions investigated and mirrored in the two national samples. Zoos could use these results to reflect on the reasons for this difference and how to intervene to increase the positive perception of female visitors on these key drivers.

In addition, the results suggests that Germans are more likely to have higher familiarity with zoological institutions. This is consistent with the fact that in countries like Germany, zoos are often public institutions, perceived as part of the social fabric, and frequented by all social classes. Not surprisingly, German zoos are the most visited in Europe [57]. 

Additionally, results showed a direct correlation between zoo familiarity and visitors’ age with emotional appeal and ethical responsibility. From the theoretical point of view, it is an important result for future studies on the topic because it rules out independent variables to be included in the next analytical model and differentiates for the specific research area. These findings suggest that familiarity with zoos, especially when cultivated over the years, as it may happen in older visitors, creates an emotional bond with these institutions that increases confidence that zoos act with ethical responsibility, thus improving their reputation. 

The fact that emotional appeal showed a correlation with zoo familiarity is also particularly relevant. Although emotions are short-lived and context-specific, several studies claimed that they influence customers in creating their opinion on the reputation of a corporation [61,62]. Moreover, the results of the ZERS trial showed a positive—even if moderate—correlation between ethical responsibility and emotional appeal. Consequently, zoo managers should give special consideration to the fact that positive emotions experienced during a visit can influence the visitor’s opinion about the reputation of that zoo as an ethical institution. To this end, zoo managers should pay special attention to explicit wildlife conservation efforts carried out by the zoo and promote emotionally engaging educational activities for visitors.

Furthermore, the analysis of the results of the individual ZERS items (see attachment 2) appeared promising, showing how zoos and their stakeholders can identify specific criticalities. For example, regarding the driver of zoo performance to question No. 18, “Do zoos educate their visitors about wildlife conservation?”, several respondents answered that they strongly disagreed and disagreed or had no definite opinion on the statement (in Italy, 11% of respondents strongly disagreed or disagreed and 21% neither agree nor disagree, while in Germany 17% strongly disagreed or disagreed and 16% neither agreed nor disagreed). Similarly, regarding question No. 19, “Zoos do scientific research?”, 31% of Italians and 43% of Germans showed that they had no definite opinion. On the other hand, visitors’ opinions in the two zoos regarding question No. 21, “Do zoos engage in nature conservation projects,” differed, with the majority of Italians (75%) agreeing or very much agreeing compared to Germans (34%) and, interestingly, with more than half of Germans (54%) having no definite opinion. 

Analysis of responses to individual ZERS items can enable the zoo to highlight areas it can work on to improve its reputation. If the zoo has received a negative response on a specific item, it can use the result to assess whether this is due to an actual deficiency in that aspect or if, despite its correct actions, there is still a misperception by the public. For example, the above-mentioned responses highlight weaknesses in communication since most zoos spend money, make significant efforts, and employ staff dedicated to scientific research and conservation projects. Still, several visitors seem not to be aware of it. This is also confirmed by the answers to question No. 22, which suggest that many visitors still think that zoo animals are taken from the wild: “Always” for 1% of Italians and 0% of Germans; “Very often” for 8% of Italians and 12% Germans; “Sometimes” for 17% of Italians and 29% of Germans; and with 26.8% of Italians and 19. 40% of Germans “not knowing”. Moreover, regarding the opinion on whether zoos are committed to maintaining animals to high welfare standards (item No. 17), 11% of respondents in Italy and 6% in Germany disagree or strongly disagree, and, remarkably, 29% of respondents in both countries do not have a definite opinion. All these features can significantly influence the reputation of zoos and the credibility of these institutions as agents of biodiversity protection, and when visitors were asked if they had negative feelings toward zoos, 50% of Italians and 38% of Germans agreed or very much agreed.

Additionally, when analyzing the answers concerning the fact that zoos act transparently and ethically, in question No. 23, a difference between the opinions of the respondents in the two countries (41% of the Italians and 22% of the Germans agree or very much agree) was highlighted. Again, 51% of Italians and 57% of Germans did not express a definite opinion. These results are particularly relevant because they show that, in the two zoos, a high percentage of visitors still need to form an opinion, and zoos should implement their actions on them. Notably, when asked directly whether they thought zoos had a good reputation, 42% of Italians and 41% of Germans respondents did not express a definite opinion, and only 37% of Italians and 30% of Germans agreed or strongly agreed. 

These responses show how significant it is for zoos to work on their reputation and how much work on this aspect needs to be done.

As shown above, the use of ZERS can provide zoos with several types of important information that may be relevant to finding strategies to improve the relationship between zoos and their stakeholders. Stakeholder management is an essential component of any business strategy in general, but it has only recently begun to be applied to zoological institutions. ZERS can be used not only to record and assess stakeholder opinions toward zoos but also to enable a more comprehensive understanding of the underlying reputational factors that elicit emotional attachment to zoological institutions. In addition, through analysis of simple descriptive statistics of individual items, the tool can be used to be focused on identifying specific critical issues that negatively influence visitor opinions. However, further applications are needed to better assess how much the type of visitors to different zoos, countries, and cultural contexts influence the response recorded. 

However, the outcomes of the first trial of ZERS questionnaire in two different European zoos showed that the tool helps investigate visitors’ opinions on the drivers that can influence the reputation of zoos, and the information collected will be useful to refine the measurement tool.

### Strengths and Limitations of the Tool and Future Developments

Reliability and validity analysis of the first trial of ZERS showed coherent and consistent evidence of its usefulness to assess the opinion of zoo visitors on the critical drivers that can determine the reputation of zoos on specific aspects of their activities and their ethical reputation. However, there are some study limitations to take into consideration.

Firstly, regarding the sample. Although the number of respondents was adequate for the study, considering that participants were not randomly selected and the questionnaire was administered only in one zoo in Italy and one in Germany, the results cannot be representative of the opinions of the entire reference population. However, this first trial of ZERS was useful in highlighting some critical issues, such as the length of the questionnaire. This has led to a revision, which is still in progress, to reduce the number of items and reword those difficult for respondents to understand. After the revision, a wider sample will be necessary to correctly test the structure of the constructs (drivers or latent variables) included in the questionnaire. Moreover, to further improve this measurement tool, validating the questionnaire on zoo visitors in different countries will be crucial.

Finally, it should be considered that ZERS was designed to evaluate the opinion of only one of the stakeholders of a zoo—its visitors—but in reality, the reputation is a multi-dimensional construct that reflects the unique dimensions on which individual stakeholders base their judgments of an institution [63]. Therefore, for a more comprehensive analysis —which would allow a zoo to better assess all the critical aspects that affect its reputation— it could be useful, in the future, to improve the tool in a way that may include the opinions also of other stakeholders (e.g., zoo worker zoological operators, environmentalists, local authorities, etc.).

Among the stakeholders, those who must be given special consideration are children. Indeed, children are perhaps the most important users of zoos, to whom the majority of the educational activities that zoos offer are dedicated. It would be very interesting for zoos to analyze children’s opinions about their reputations. However, for this purpose, it will be necessary to design a suitable version of ZERS questionnaire. Specifically, the ZERS items will have to be adapted in number and wording to make them understandable to a younger audience. 

Additionally, it will also be helpful to administrate the ZERS questionnaire to assess the opinion of non-visitor population, considering that almost no research exists to date comparing visitors versus non-visitors on many zoo-related topics. This would be of particular interest because it would help to explain if the ethical reputation of zoos can influence the propensity to visit zoos. Therefore, analyzing this population’s opinion could help zoos find strategies to expand their visitor base.

In the future, the ZERS questionnaire presented can be integrated with other measurement tools to investigate other stakeholders’ opinions. However, at this first stage, it was decided to analyze the opinion about the ethical reputation of zoos only in the category of young and adult visitors, who do not represent all stakeholders but are among those who very easily can spread word of mouth about the reputation of a particular zoo.

However, a very important step was represented by the identification of the main drivers that can impact the reputation of zoos. Based on them, it will then be possible to customize ZERS, creating questionnaires with items adapted to analyze the opinions on the reputation of zoos—determined by the particular interests of each stakeholder—of different stakeholder categories. The results will provide important information to the zoo on what it has been able to communicate regarding its efforts for animal welfare, its work in biodiversity conservation, and its ability to implement effective educational projects. This can allow the individual zoo to figure out what aspect to improve. Furthermore, this will allow the zoo also to choose different strategies to satisfy that particular stakeholder category that has underlined a possible critical issue. This information can then be shared with other zoos to benefit the community of zoos as a whole.

## 5. Conclusions

This paper reported the development of a tool, ZERS, that assesses and highlights people’s perceptions about 12 drivers that can influence the reputation of zoos. Similar tools, such as Reputation Quotient ^(SM)^, are well established for the evaluation of the reputation of other corporations [63], but, to our knowledge, there are no similar tools to evaluate the reputation and ethical aspects of zoological institutions. Yet, nowadays, zoos are progressively under the scrutiny of public opinion, and many factors can negatively influence their reputation by offering an excuse to those who consider these institutions obsolete or a “nineteenth-century anachronism” [4].

In the development of the tool, many drivers that can influence the reputation of a zoo have been identified. Zoos must be very careful of their reputation to ensure that they thrive in the future as major conservation organizations, as a negative reputation may quickly lead to a reduction in the number of visitors and funding for conservation projects [58,64]. Reputation can take a long time to build up and coalesce in people’s minds, but research shows that it can be extremely difficult to change once formed [65]. This must be taken into adequate consideration, especially with regard to a negative reputation. Therefore, there is an urgent need to develop tools to analyze visitors’ opinions on components that can affect the reputation of zoos.

So far, despite the vast literature on the reputation assessment of companies whose main objective is to improve their income, there is no research on the development of tools to assess the reputation of zoos. This is probably due to the fact that zoos—which have as their goals not only economic interests but also, and above all, the welfare of wild living beings, the protection of biodiversity, and the education of their visitors—are much more complex entities. ZERS can fill this gap and help these institutions to assess their ethical reputations. Zoological associations know very well how important it is for them to act ethically not only in the management of animal welfare but also in their actions and communication with all other stakeholders. For this reason, in 1995, the World Association of Zoos and Aquaria (WAZA) drew up its own ethical code, which has been continuously adapted and updated over the years and to which all its members must adhere. 

ZERS can help zoological associations evaluate how much the public perceives the commitment of their members. At the same time, the use of ZERS can also enable individual zoos to highlight critical issues and implement strategies to improve them. By addressing them, zoos can not only increase people’s trust and involvement in their biodiversity conservation efforts but also, by reflecting on measurable parameters, they are encouraged to operate as ethical institutions, “ethical arks” committed to advancing higher standards and practices towards all their stakeholders.

## Figures and Tables

**Table 1:** ZERS questionnaire layout with items for each specific drive. The complete questionnaire is available in supplementary materials (S1).

Drivers Category	Specific Driver	N.	Item
FUNCTIONAL DRIVERS	PERFORMANCE (PERF)	17	Zoos are committed to guaranteeing high standards of animal welfare
		18	Zoos educate their visitors about wildlife conservation
		19	Zoos do scientific research
		21	Zoos dedicate themselves to conservation projects in the wild
		31	Zoos are going to become a bigger reality in the future
	PRODUCT AND SERVICE (PR_SR)	12	Zoos enable a direct experience of wild animals
		20	The time spent in zoos is a good value for the money spent on the ticket
		27	Zoos’ staff helped me in having a nice day at the zoo
	WORKPLACE (WORKP)	24	Zoos’ staff is passionate about their job
		25	Zoos are well managed
		26	Zoos are good companies to work for
MOTIVATIONAL DRIVERS	VISION (VISION)	34	Zoos make unclear and undefined promises
		35	Zoos have excellent management
		36	Zoos clearly explain their goals and their mission
		34	Zoos make unclear and undefined promises
		35	Zoos have excellent management
	ETHICAL RESPONSIBILITY (ETR)	23	Zoos act in a transparent and ethical way
		33	Zoos are open and transparent about the way they operate
		37	Zoos are accurate when disseminating information
		38	Zoos do what they say they are going to do
		39	Zoos are dishonest and false in their communications
	SOCIAL RESPONSIBILITY (SOCRES)	29	Zoos are environmentally responsible organizations
		30	Zoos support good causes
		32	Zoos handle their animals in a responsible way
RELATIONAL DRIVERS	FAMILIARITY (FAM)	1–5	How many times have you visited the following facilities in the last 12 months? Zoos Aquariums Natural parks and reserves Safari parks Other facilities that house wild animals
		6	Rate your degree of familiarity with zoos
	EMOTIONAL APPEAL (EMA)	8	I trust zoos
		9	I have negative feelings towards zoos
		10	Zoos have a good reputation
		11	I admire and respect zoos
		13–16	How frequently do you feel each of these emotions when thinking about animal extinctions? Worried Alarmed Unconcerned Hopeful
		52	I will leave feedback about how the zoo can be improved
		53	If a zoo has to face a problem, I trust it will make the right choice
	LOYALTY (LOY)	7	Do you have a season ticket or a membership pass for a zoo?
	INTENTION TO PURCHASE (ITP)	49	What’s the likelihood that you will visit zoos in the future?
THIRD-PARTY INFLUENTIAL DRIVERS	KNOWLEDGE (KNOW)	22	Are animals in zoos taken from the wild?
	POSITIVE WORD OF MOUTH (PWM)	50	I will suggest to a friend to go to zoos
		51	I will say positive things about zoos

**Table 2:** Reliability Scale of ZERS drivers. Crombach’s α CI values ranging from 0.70 to 0.85 are considered acceptable.

		95.0% Confidence Interval	
	Cronbach’s α	Lower	Upper
Ethical responsibility	0.848	0.812	0.870
Familiarity	0.694	0.616	0.734
Loyalty	0.148	0.080	0.391
Workplace	0.703	0.634	0.757
Performance	0.754	0.705	0.797
Social responsibility	0.754	0.702	0.802
Emotional appeal	0.767	0.712	0.805
Extinction awareness	0.696	0.643	0.763
Vision	0.675	0.60	0.736

**Table 3:** Correlation matrix. The correlation matrix was used to test the hypothetical relationship pattern among selected key drivers. The results provide indications of statistically significant correlation between Ethical responsibility with all other variables, moderate positive correlation with Emotional appeal, r (263) = 0.581, *p* < 0.01, and small correlation with Familiarity zoo-related r (239) = 0.133, *p* < 0.05 and Familiarity not zoo-related r (235) = 0.148, *p* < 0.05. * Pearson Correlation *p* < 0.05 level (2-tailed). ** Pearson Correlation *p* < 0.01 level (2-tailed).

	ETR	EMA	FAM_ZOO	FAM_NOZOO
ETR	1			
EMA	0.581 **	1		
FAM_ZOO	0.133 *	0.164 *	1	
FAM_NOZOO	0.148 *	0.053	0.335 **	1

**Table 4:** Mann–Whitney test parameters for selected questionnaire items. Example of the item coding system: QXX_ETR = Q (question) × X (item order in the questionnaire), _ETR (item-related driver). For the Mann–Whitney test, the location parameter is given by the Hodges–Lehmann estimate. Levene’s test is significant (*p* < 0.05), suggesting a violation of the equal variance assumption (it may determine a bias in the interpretation).

	W	P	Hodges–Lehmann Estimate	Rank-Biserial Correlation
Q24_WORKP	9444.50	0.04	3.15 × 10^−5^	0.14
Q23_ETR	10,399.50	9.71 × 10^−5^	5.91 × 10^−5^	0.26
Q6_FAM	4798.00	2.06 × 10^−5^	−1.00	−0.31
Q10_EMA	9168.00	0.09	4.74 × 10^−5^	0.12
Q11_EMA.	9526.50	0.03	4.22 × 10^−5^	0.16
Q51_PWM	11,299.00	9.96 × 10^−8^	1.00	0.38
Q18_PERF	8782.50	0.37	1.34 × 10^−5^	0.06
Q19_PER	8834.00	0.29	1.65 × 10^−5^	0.07
Q21_PERF	11,817.50	3.36 × 10^−10^	1.00	0.44

**Table 5:** Independent Samples T-Test on the differences between nationality on the relevant constructs checked with gender as a grouping variable. For the Student *t*-test, the effect size is given by Cohen’s d. For the Student *t*-test, the location parameter is given by the mean difference.

	t	df	*p*	Mean Difference	SE Difference	95% CI for Mean Difference		Cohen’s d
						Lower	Upper	
FAM	2.090	215	0.038	−0.950	0.454	1.845	0.054	−0.299
ETR	4.928	265	<0.001	2.112	0.429	1.268	2.956	0.640
EMA	4.005	268	<0.001	1.117	0.279	0.568	1.666	0.517
PWM	5.65	271	<0.001	2.04	0.36	1.33	2.76	0.73
WORK	2.79	271	<0.001	0.65	0.23	0.19	1.10	0.36
PERF	5.21	268	<0.001	1.99	0.39	1.22	2.75	0.66

**Table 6:** Differences between nationality and gender on familiarity with visitors. The analysis was conducted with ANOVA Type III Sum of Squares.

Cases	Sum of Squares	df	Mean Square	F	*p*
GENDER	16.317	1	16.317	1.654	0.200
NATIONALITY	2.304	1	2.304	0.234	0.629
GENDER × NATIONALITY	48.801	1	48.801	4.946	0.027
RESIDUALS	2091.737	212	9.867		

**Table 7:** Post Hoc Comparisons of gender respondents in the zoos. The *p*-value was adjusted for comparing a family of four using Tukey’s correction.

		Mean Difference	SE	t	*p* _tukey_
Male, Italian	Female, Italian	0.542	0.528	1.026	0.734
	Male, German	1.006	1.025	0.981	0.760
	Female, German	−1.022	0.560	−1.826	0.264
Female, Italian	Male, German	0.464	1.011	0.459	0.968
	Female, German	−1.564	0.533	−2.935	0.019
Male, German	Female, German	−2.028	1.028	−1.973	0.201

**Table 8:** Results of the multiple regressions applied to predict differences in emotional appeal (EMA) from gender, age, and education level (EDL), pet ownership (PETOWN), urbanization (URBANIZ), income level (INCOME), and zoo familiarity (FAM-ZOO). In the table the *p*-values < 0.05 indicate the corresponding variable is a statistically significant predictor of the outcome variable.

Emotional Appeal	B	SEB	β	t	Sig.
Gender	−0.115	0.313	−0.025	−0.369	0.713
AGE	0.464	0.163	0.204	2.854	0.005
EDL	−0.269	0.246	−0.076	−1.092	0.276
PETOWN	−0.014	0.299	−0.003	−0.047	0.963
URBANIZ	−0.152	0.167	−0.061	−0.910	0.364
INCOME	−0.018	0.087	−0.014	−0.207	0.836
FAM_ZOO	0.229	0.095	0.161	2.417	0.016

**Table 9:** Results of the multiple regressions applied to predict differences in ethical responsibility (ETR) from gender, age, and education level (EDL), pet ownership (PETOWN), urbanization (URBANIZ), income level (INCOME), and zoo familiarity (FAM-ZOO). In the table the *p*-values < 0.05 indicate the corresponding variable is a statistically significant predictor of the outcome variable.

Ethical Responsibility	B	SEB	β	t	Sig.
Intercept	20.087	1.598		12.568	0.000
Gender	−0.287	0.491	−0.040	−0.585	0.559
AGE	0.852	0.257	0.237	3.310	0.001
EDL	−0.350	0.383	−0.064	−0.914	0.362
PETOWN	−0.222	0.466	−0.032	−0.476	0.634
URBANIZ	−0.328	0.268	−0.082	−1.223	0.223
INCOME	−0.270	0.137	−0.133	−1.975	0.050
FAM_ZOO	0.294	0.149	0.132	1.980	0.049

## Data Availability

The data presented in this study are available on request from the corresponding authors.

## Supplementary Materials

The following supporting information can be downloaded at: https://www.mdpi.com/article/10.3390/ani12202802/s1, File S1: ZERS questionnaire; File S2: Descriptives and survey plots.
