# Supplementary material for: Development of A Tool for Assessing the Reputation of Zoos: The Zoo Ethical Reputation Survey (ZERS)
Source: Animals (Basel). 2022 Oct 17;12(20):2802. doi: 10.3390/ani12202802 (PMC9597707; doi:10.3390/ani12202802)
Supplement: Supplementary file 1 [file animals-12-02802-s001.zip › S1.pdf]

Supplementary File S1

THE ZERS QUESTIONNARIE

How many times have you visited the following facilities in the last 12 months? Put a cross for each row.

		Never	Once	2-3 times	4-10 times	More than 10 times
1	Zoos					
2	Aquariums					
3	Natural Parks and reserves					
4	Safari Parks					
5	Other facilities that house wild animals					

6 Rate your degree of familiarity with zoos.

None	Low	Average	High	Very high
○	○	○	○	○

7 Do you have a season ticket or a membership pass for a zoo?

- ○ Yes
- ○ No

Please indicate your opinion about the following statements.

		Strongly disagree	Disagree	Neither agree nor disagree	Agree	Strongly agree
8	I trust zoos.					
9	I have negative feelings towards zoos.					
10	Zoos have a good reputation.					
11	I admire and respect zoos.					
12	Zoos enable a direct experience of wild animals.					

How frequently do you feel each of these emotions when thinking about animal extinctions? Put a cross for each row.

		Always	Very Often	Sometimes	Rarely	Never
13	**Worried**					
14	**Alarmed**					
15	**Unconcerned**					
16	**Hopeful**					

Please indicate your opinion about the following statements

		Strongly disagree	Disagree	Neither agree nor disagree	Agree	Strongly agree
**17**	**Zoos are committed to guaranteeing high standards of animal welfare.**					
**18**	**Zoos educate their visitors about wildlife conservation.**					
**19**	**Zoos do scientific research.**					
**20**	**The time spent in zoos is a good value for the money spent on the ticket.**					
**21**	**Zoos dedicate themselves to conservation projects in the wild.**					

22 Are animals in zoos taken from the wild?

- O Always
- O Very often
- O Sometimes
- O Rarely
- O Never
- O I don’t know

Please indicate your opinion about the following statements

		Strongly disagree	Disagree	Neither agree nor disagree	Agree	Strongly agree
23	Zoos act in a transparent and ethical way.					
24	Zoos' staff is passionate about their job.					
25	Zoos are well managed.					
26	Zoos are good companies to work for.					
27	Zoos' staff help me in having a nice day at the zoo.					
28	Zoos promote projects with local communities.					
29	Zoos are environmentally responsible organizations.					
30	Zoos support good causes.					
31	Zoos are going to become a bigger reality in the future.					
32	Zoos handle their animals in a responsible way.					
33	Zoos are open and transparent about the way they operate.					
34	Zoos make unclear and undefined promises.					
35	Zoos have excellent management.					
36	Zoos explain clearly their goals and their mission.					
37	Zoos are accurate when disseminating information.					
38	Zoos do what they say they are going to do.					
39	Zoos are dishonest and false in their communications.					
40	It is meaningful that zoos make society aware of the importance of conservation.					
41	It is meaningful that zoos conserve and protect threatened animals.					
42	It is meaningful that zoos breed endangered animals.					
43	It is plausible that zoos use their animals as a learning resource.					
44	It is meaningful that zoos perform educational programmes for children.					

**45**	**It is plausible that zoos entertain visitors through various programmes.**					
**46**	**It is meaningful that zoos are a place for scientific research.**					
**47**	**It is meaningful that zoos educate about conservation issues.**					
**48**	**It is meaningful that zoos are a place for recreation.**					

On a scale of 0 to 5, with 5 being the highest, what's the likelihood that you would do the following actions? (0 is "absolutely not" and 5 is "absolutely yes")

		0	1	2	3	4	5
**49**	**I will visit zoos in the future.**						
**50**	**I will suggest to a friend to go to zoos.**						
**51**	**I will say positive things about zoos.**						
**52**	**I will leave feedback about how the zoo can be improved.**						
**53**	**If a zoo has to face a problem, I will trust it will make the right choice.**						

Only for statistical purposes, please fill the form with your personal information. The questionnaire is anonymous.

54 What is your age?

- 14 – 18 years
- 19 – 25 years
- 26 - 34 years
- 35 - 54 years
- 55 to 64 years
- over 64

55 Gender

- Male
- Female
- I prefer not to answer

56 Do you currently have pets at home?

- Yes
- No

57 Do you practice sports or activities connected to the natural world? (Trekking, mountain climbing, diving, etc.)

59 Do you have specific training on animals or nature?

- Yes
- No

60 Where do you live?

- Very rural/wilderness
- Village or rural
- Small town (under 10 000 habitants)
- Medium/big-sized town

61 Have you ever travelled only or mainly to see animals?

- Yes
- No

62 Which of these describes your income last year?

O Yes<br>O No<br><br>**58 Highest level of education completed**<br>O No schooling completed<br>O Elementary school<br>O Middle school<br>O High school<br>O University degree<br>O Postgraduate degree/PhD	O Less than 14000 €<br>O 14000 - 29999 €<br>O 30000 - 43000 €<br>O More than 43000 €<br>O I prefer not to answer
