# Supplementary material for: Development of A Tool for Assessing the Reputation of Zoos: The Zoo Ethical Reputation Survey (ZERS)
Source: Animals (Basel). 2022 Oct 17;12(20):2802. doi: 10.3390/ani12202802 (PMC9597707; doi:10.3390/ani12202802)

**Supplementary File S2**

# <u>DESCRIPTIVES AND SURVEY PLOTS</u>

## PERFORMANCE

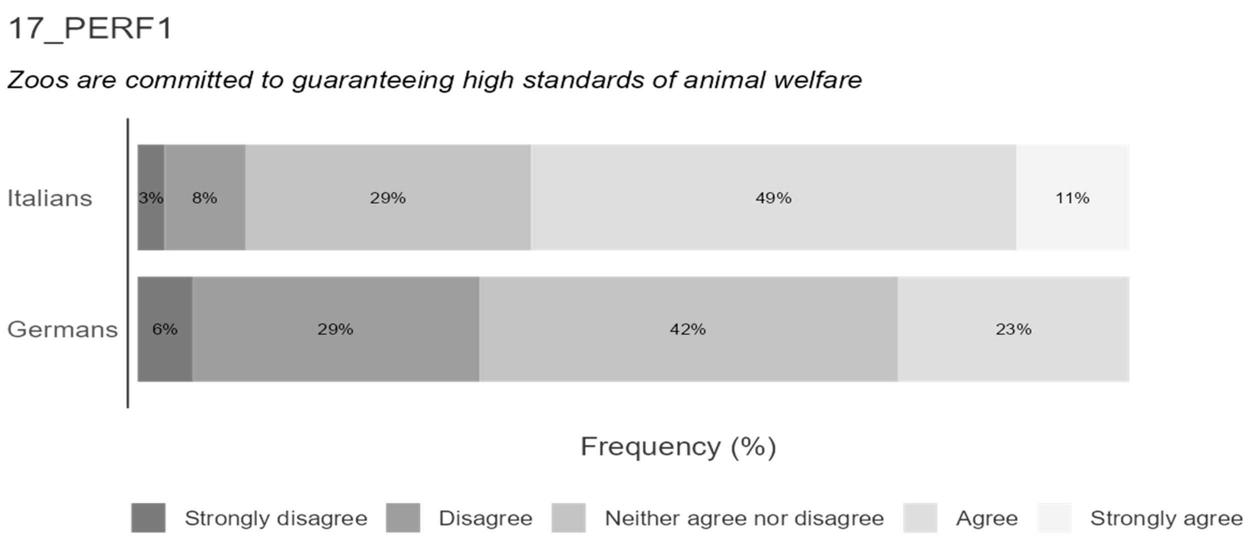


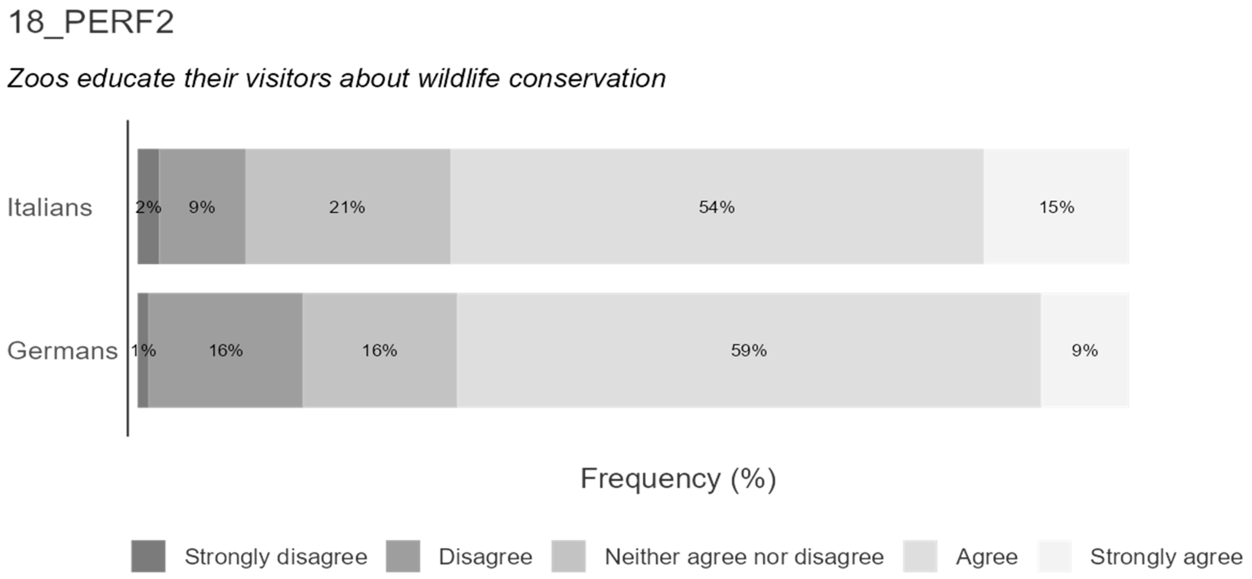

## 19_PERF3

*Zoos do scientific research*

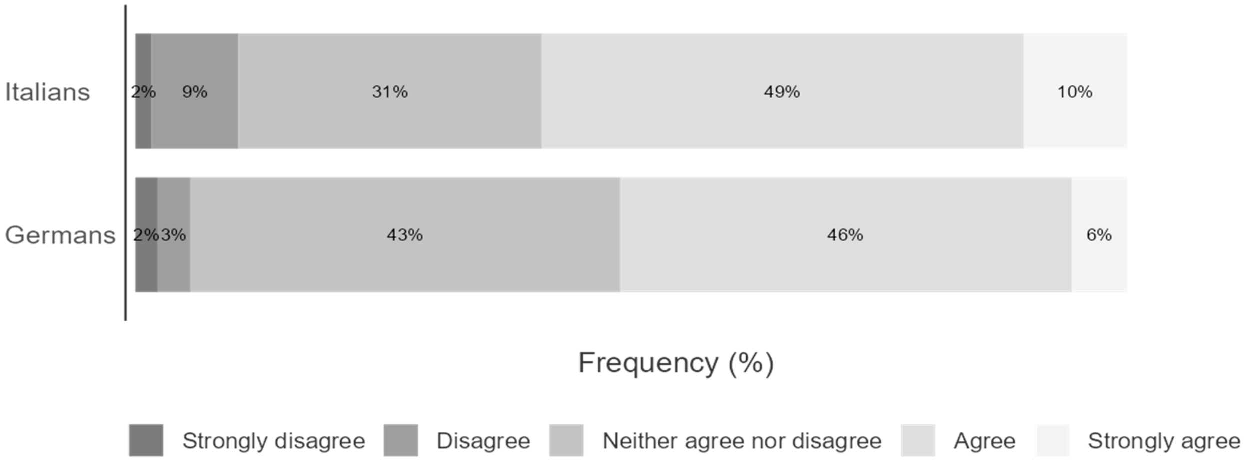


## 21_PERF4

*Zoos dedicate themselves to conservation projects in the wild*

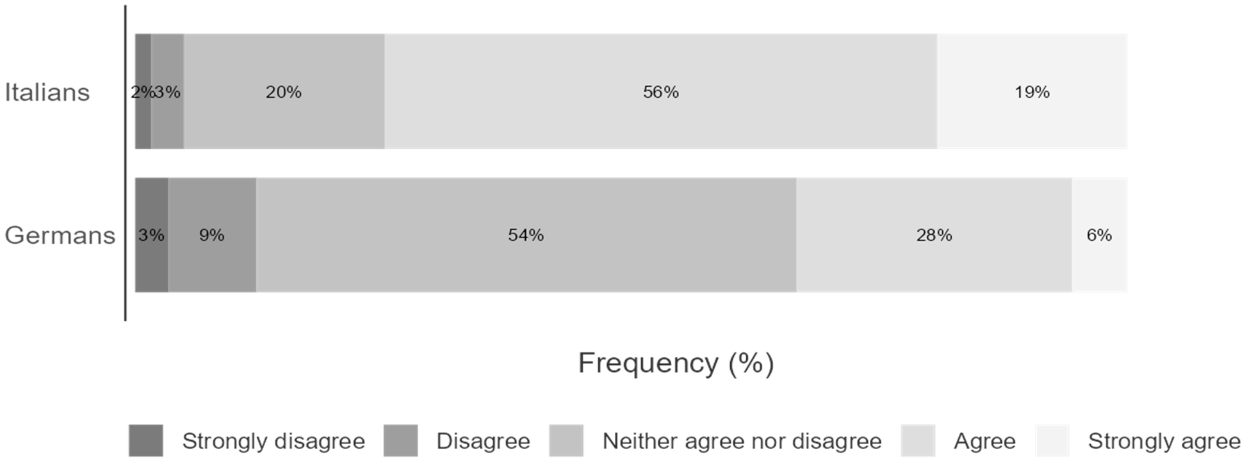


## 31_PERF5

*Zoos are going to become a bigger reality in the future*

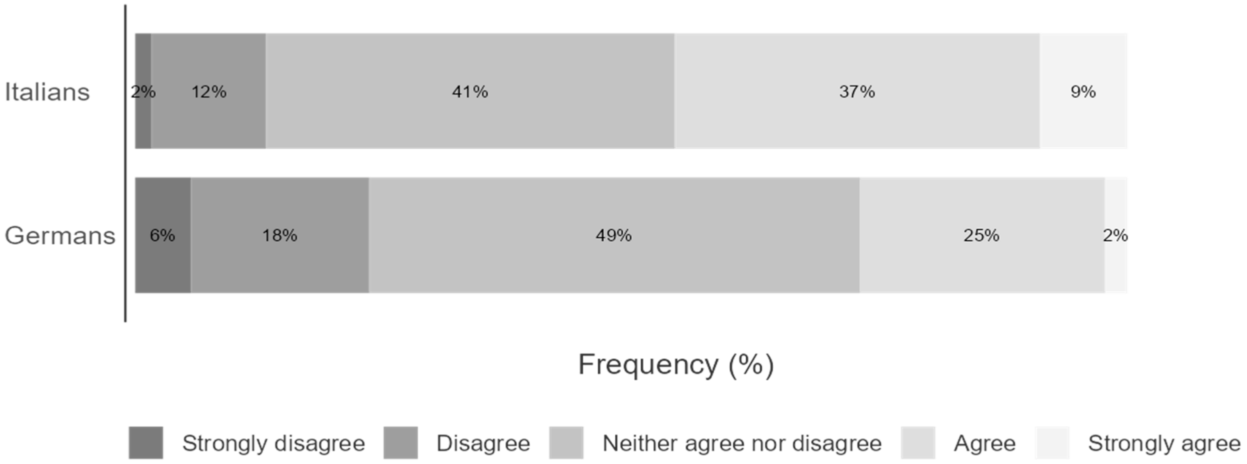

# PRODUCT AND SERVICE

20_PR_SR1

*The time spent in zoos is a good value for the money spent for the ticket.*

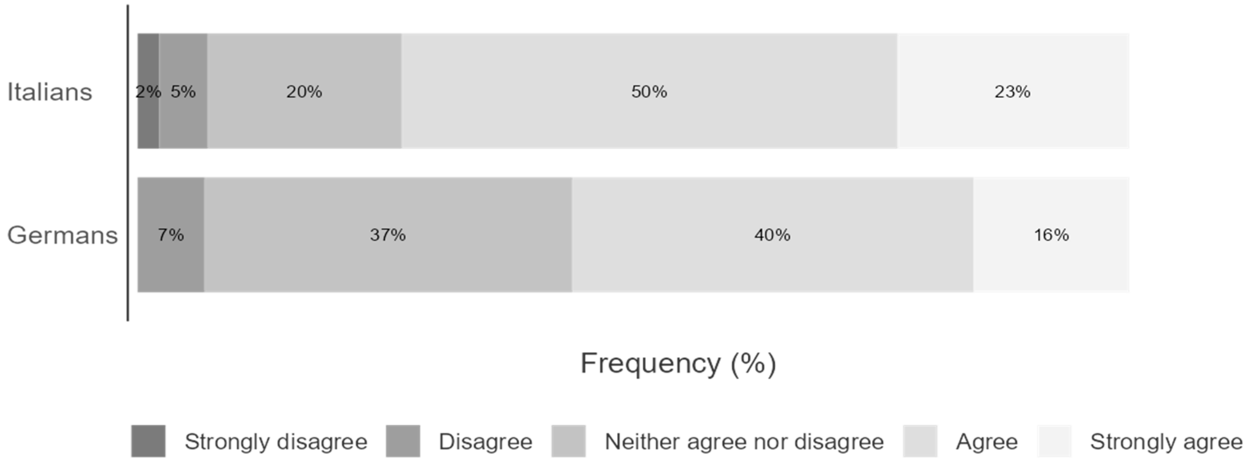


27_PR_SR2

*Zoos' staff help me in having a nice day at the zoo.*

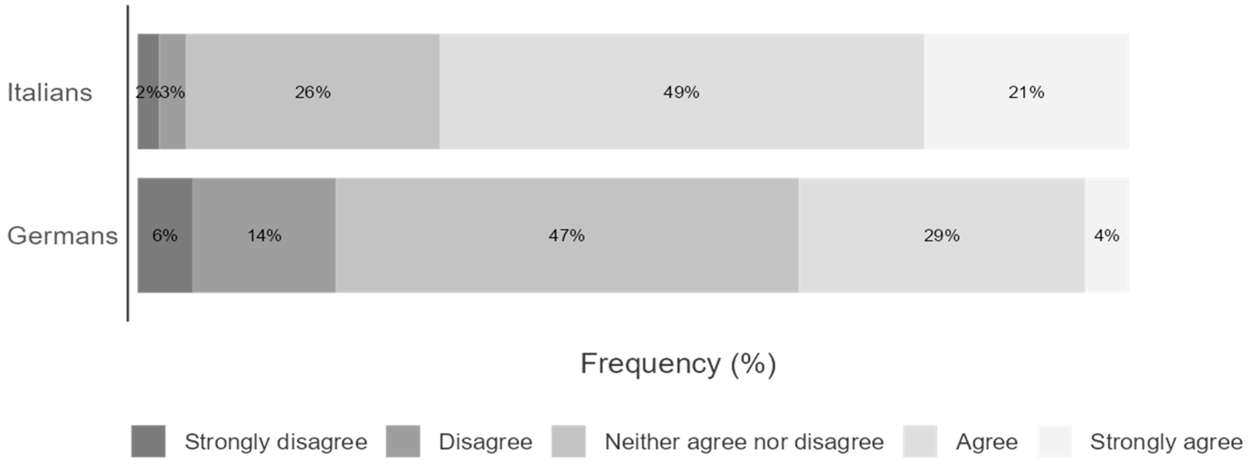

## WORKPLACE

### 24_WORKP1

*Zoos' staff is passionate to their job.*

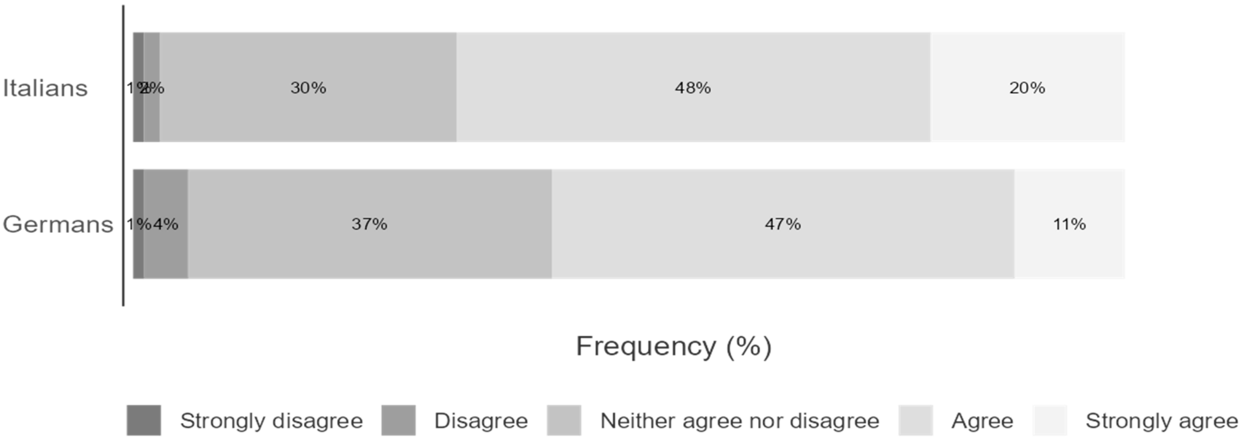


### 25_WORKP2

*Zoos are well managed.*

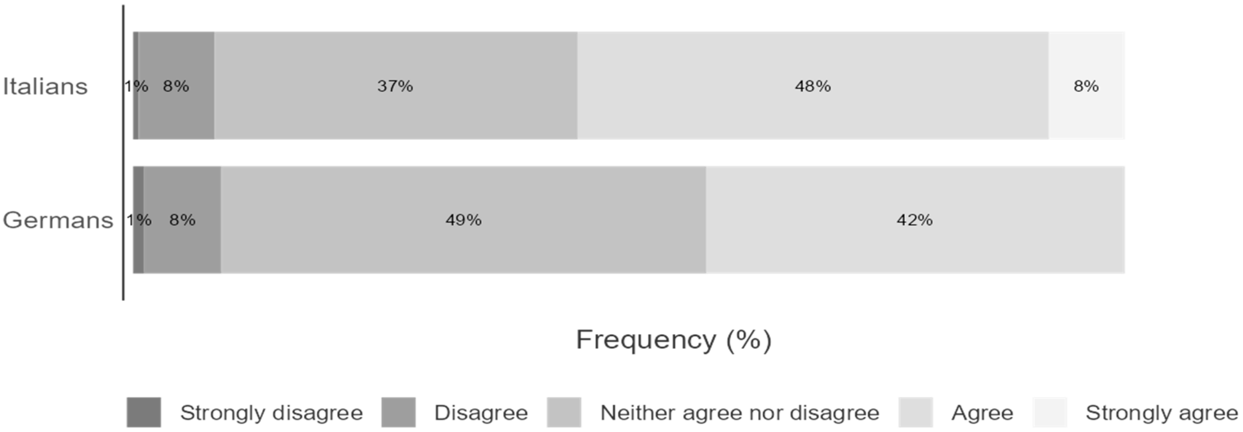


### 26_WORKP3

*Zoos are good companies to work for.*

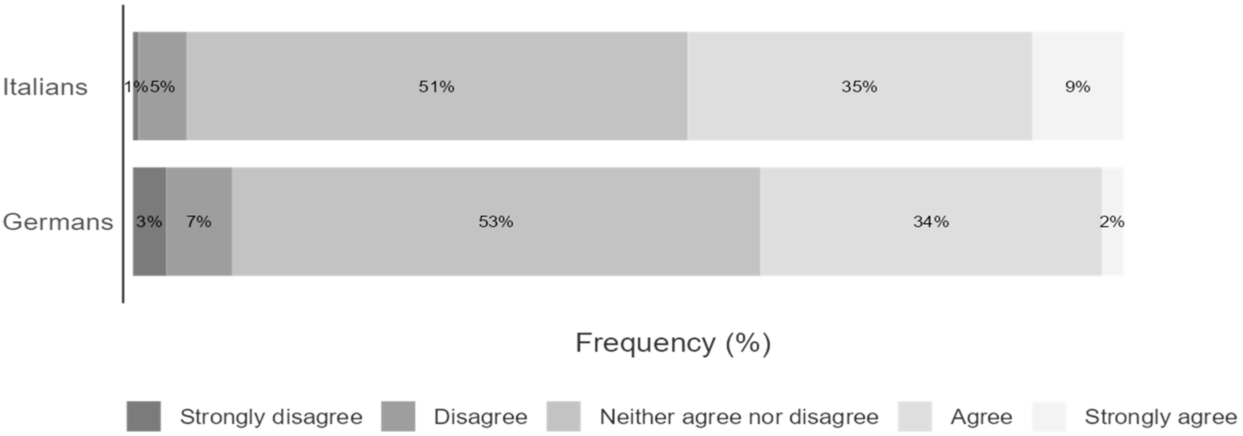

# SOCIAL RESPONSIBILITY

## 28_SOCRES1

*Zoos promote projects with local communities.*

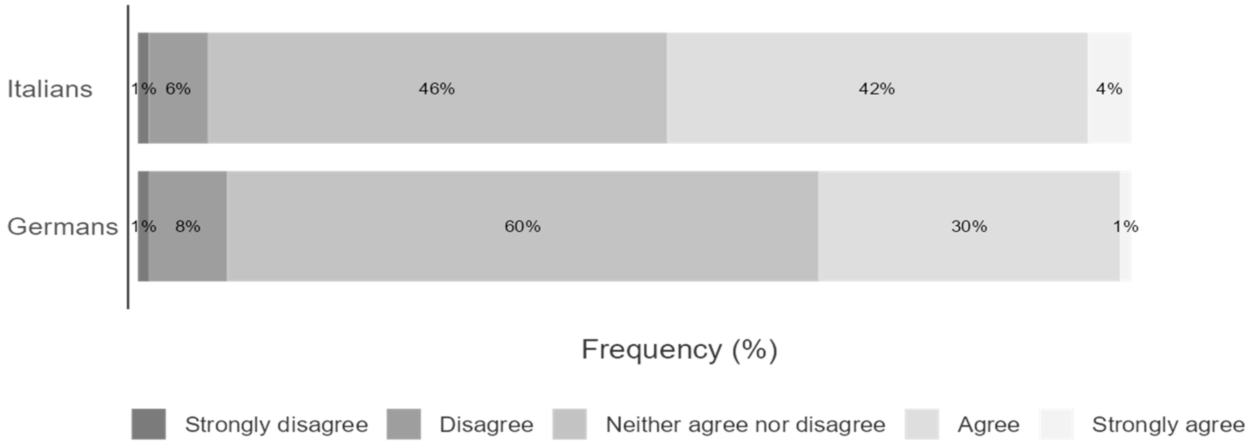


## 29_SOCRES2

*Zoos are environmentally responsible organizations.*

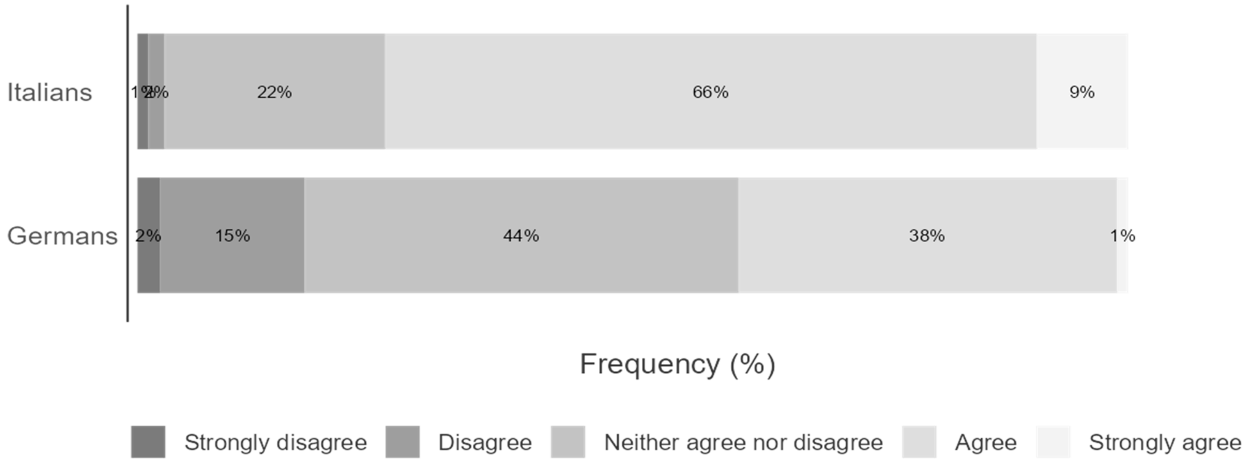


## 30_SOCRES3

*Zoos support good causes.*

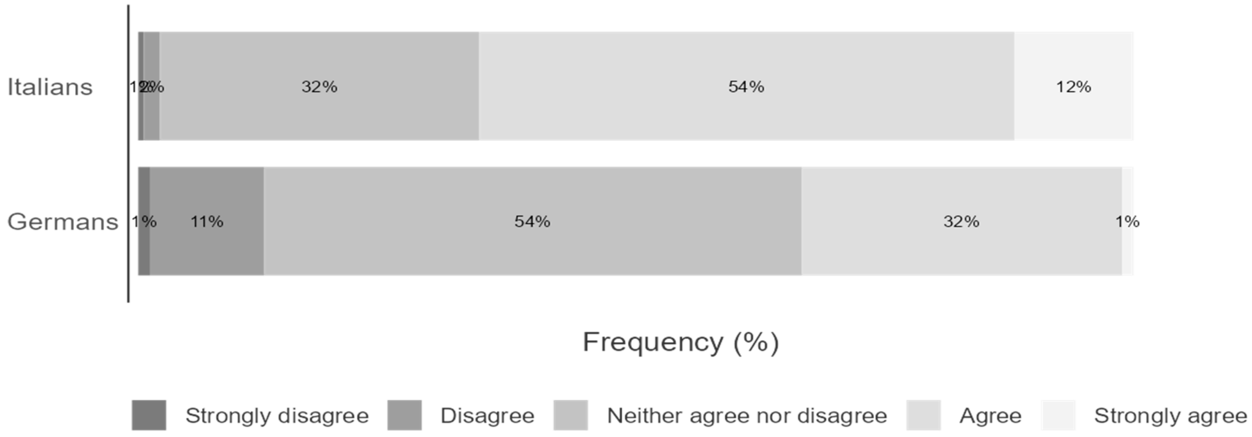

# VISION

## 34_VISION1R

*Zoos make unclear and undefined promises.*

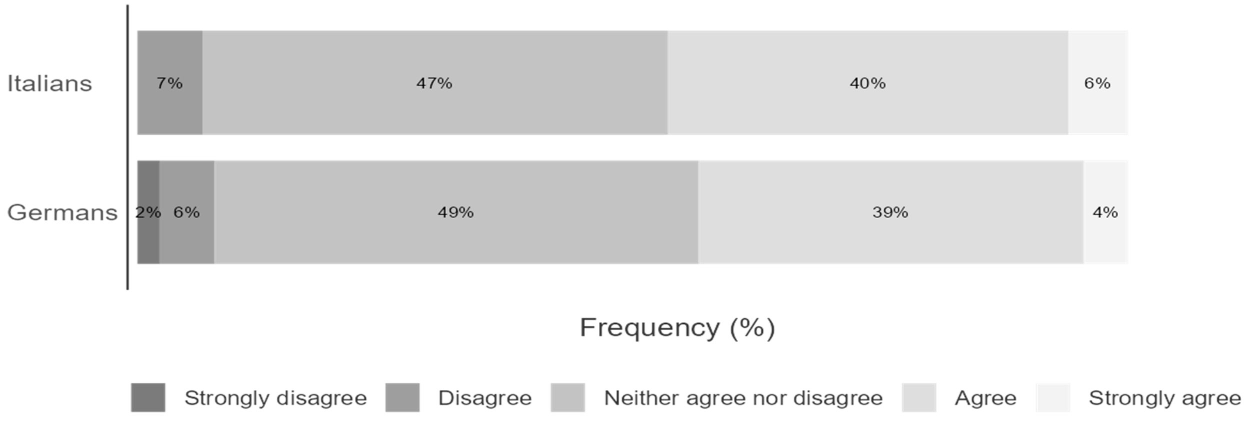


## 35_VISION2

*Zoos have excellent management.*

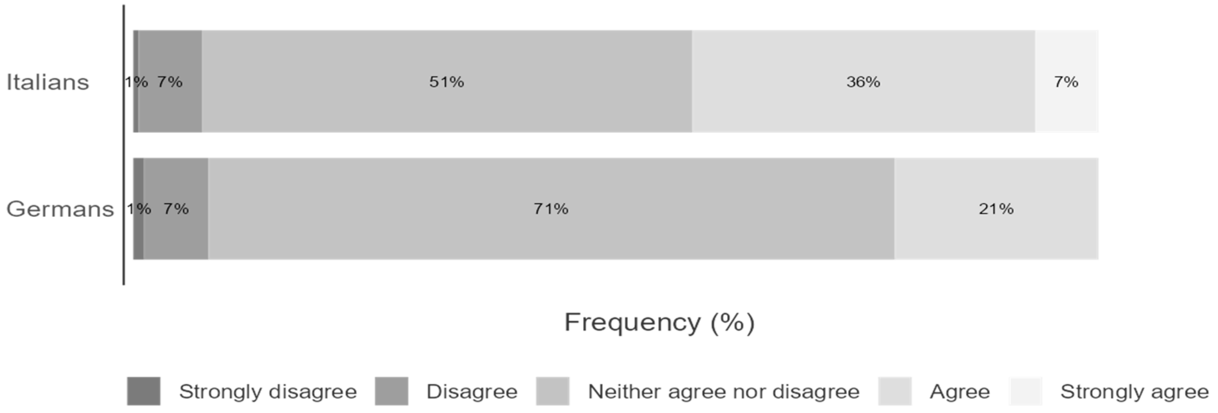


## 36_VISION3

*Zoos explain clearly their goals and their mission.*

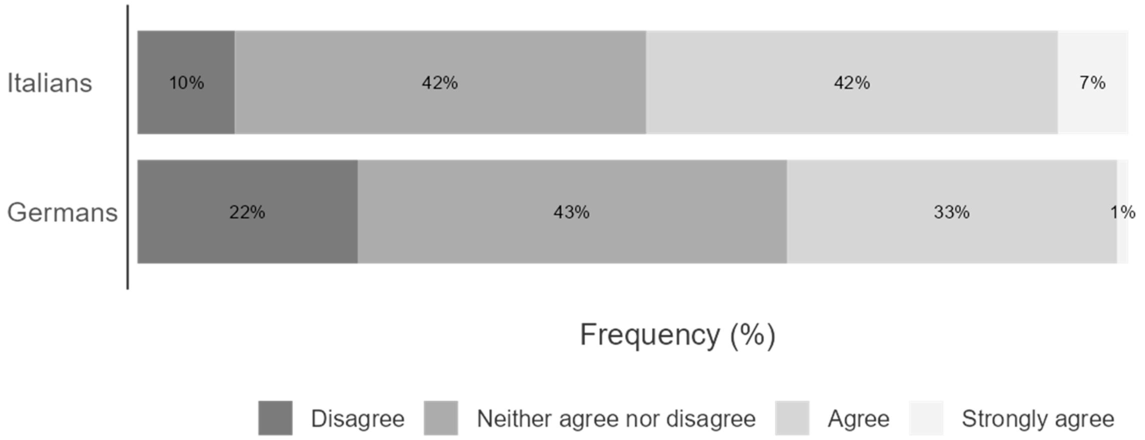

# ETHICAL RESPONSIBILITY

23_ETR1

*Zoos act in a transparent and ethical way.*

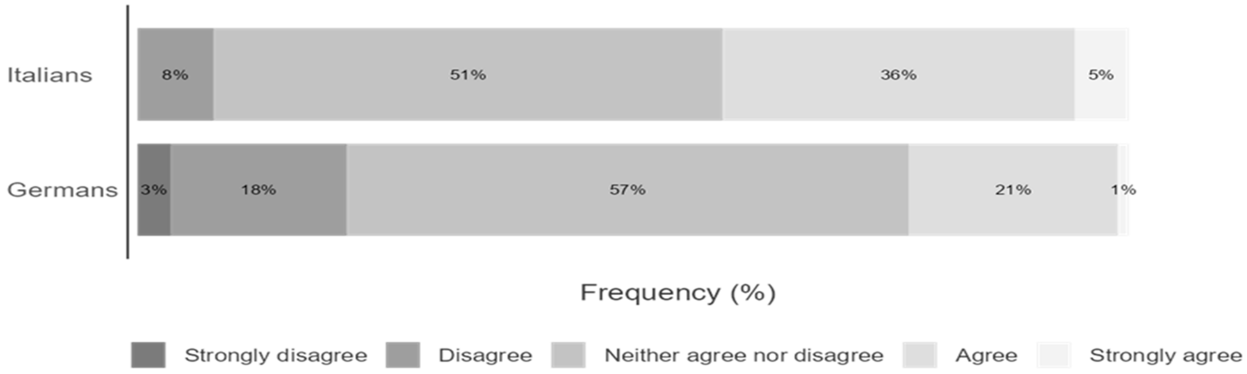


33_ETR2

*Zoos are open and transparent about the way they operate.*

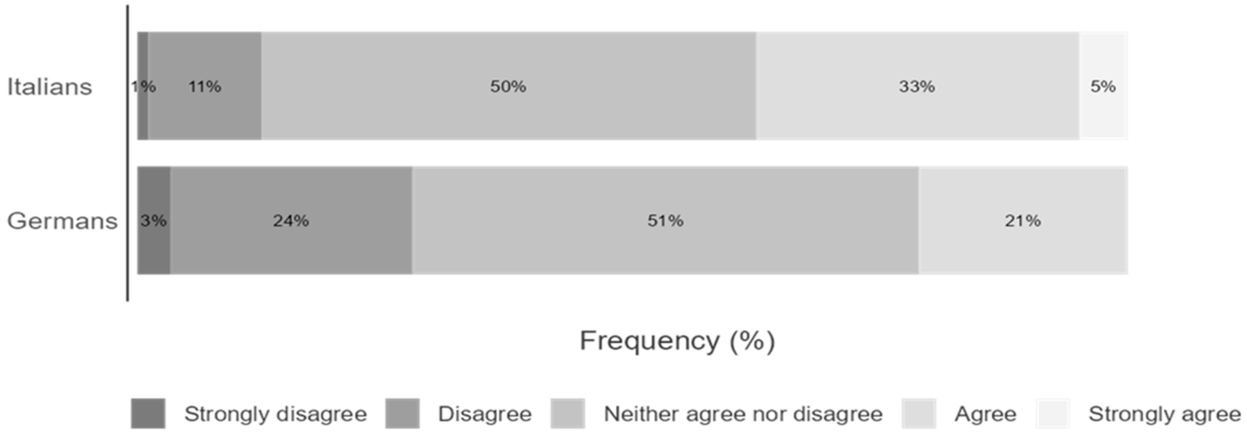


37_ETR3

*Zoos are accurate when disseminating information.*

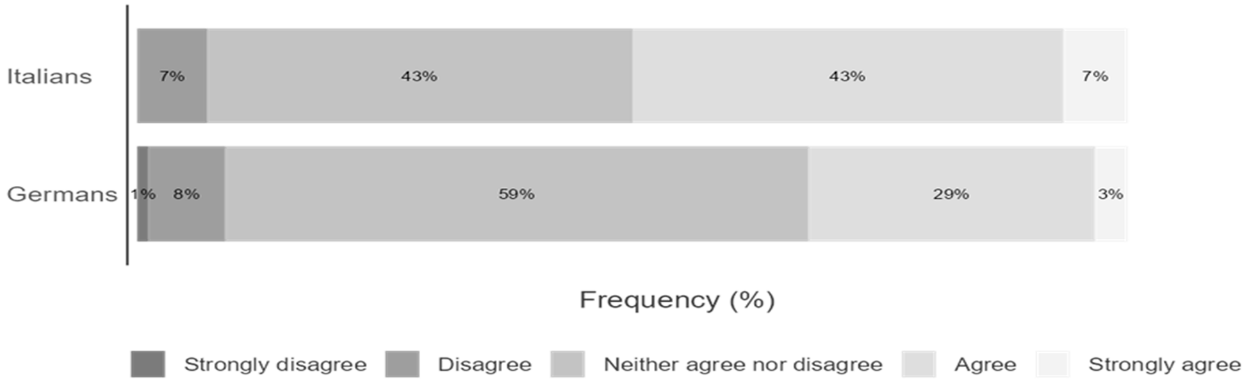

38_ETR4

*Zoos do what they say they are going to do.*

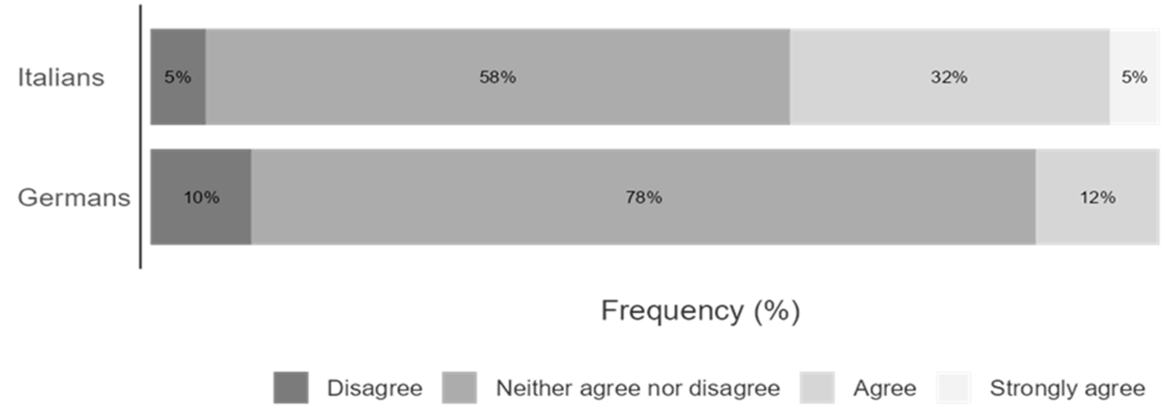


39_ETR5R

*Zoos are dishonest and false in their communications.*

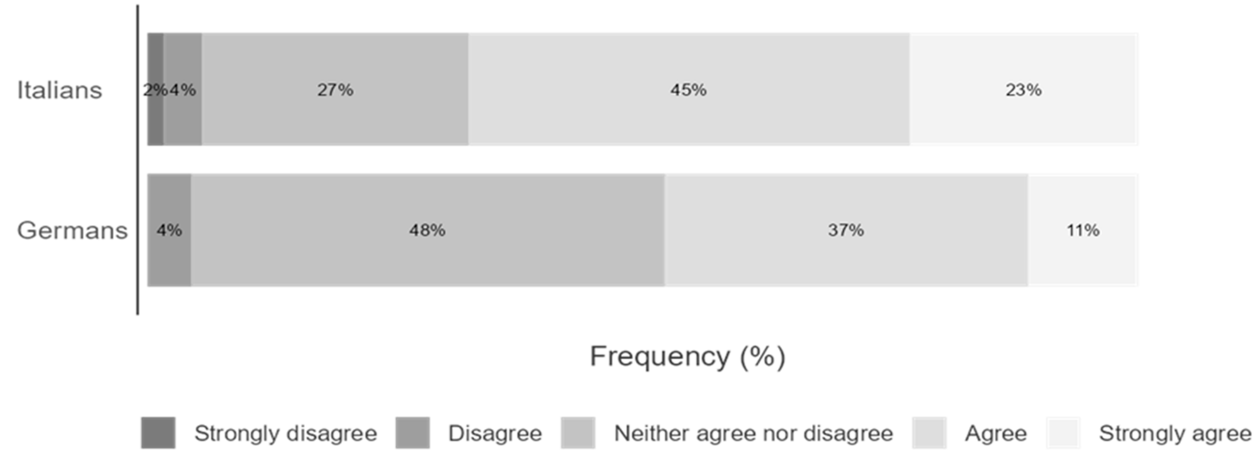


32_ETR6

*Zoos handle their animals in a responsible way*

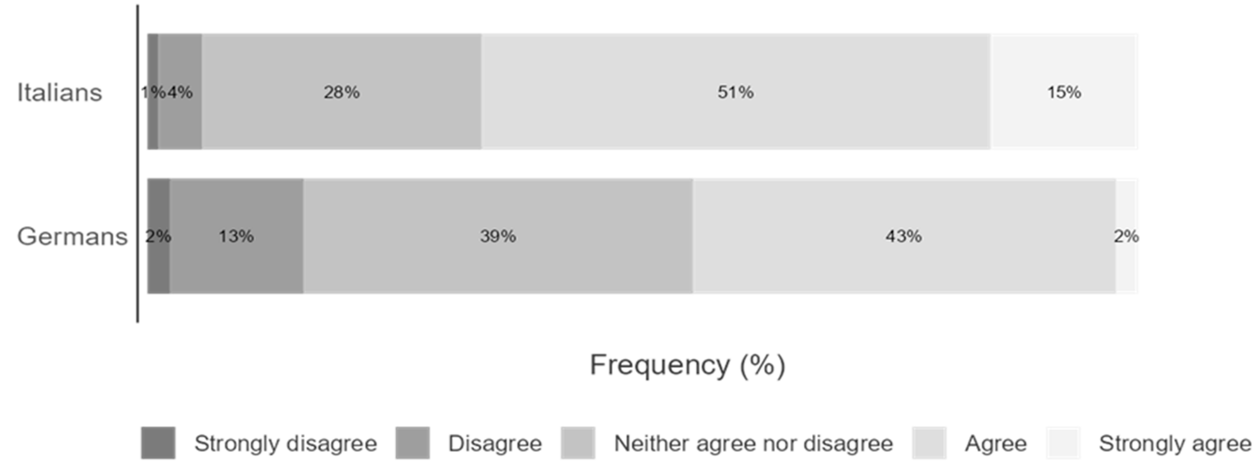

# POSITIVE WORD OF MOUTH

50_PWM1

*I will suggest to a friend to go to zoos.*

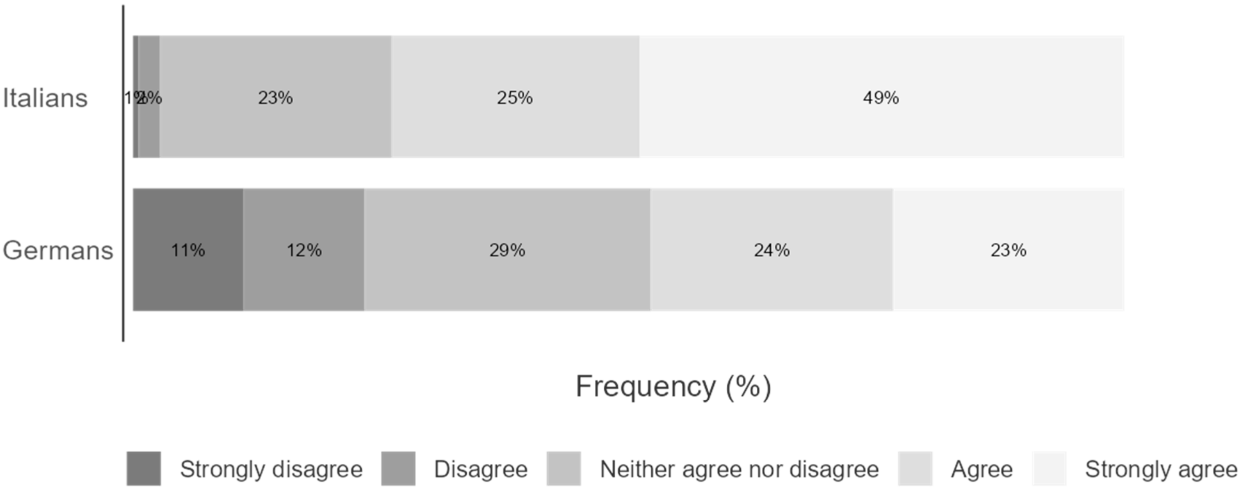


51_PWM2

*I will say positive things about zoos.*

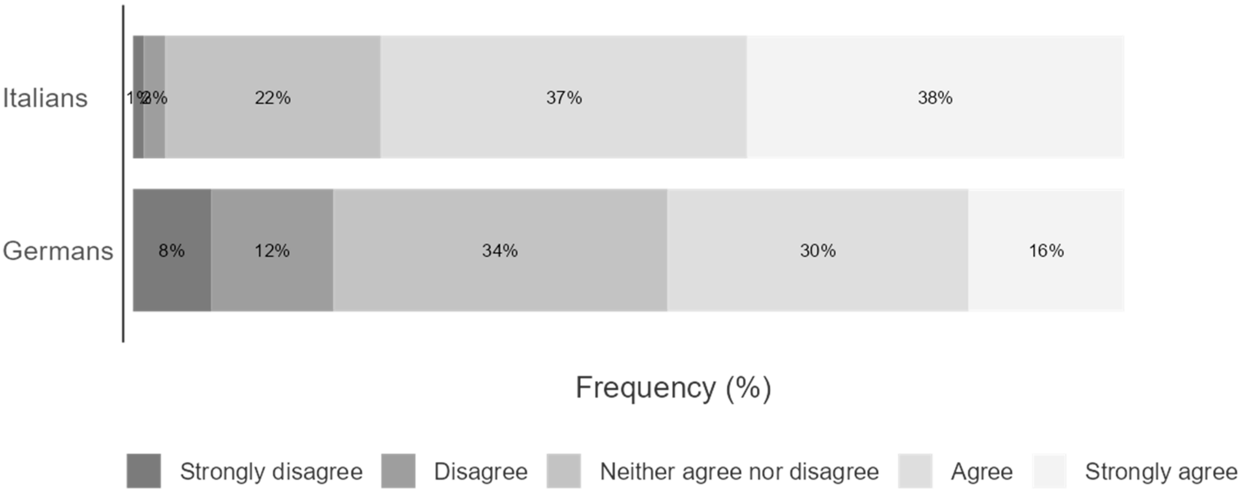

# LOYALTY

## 7_LOY1

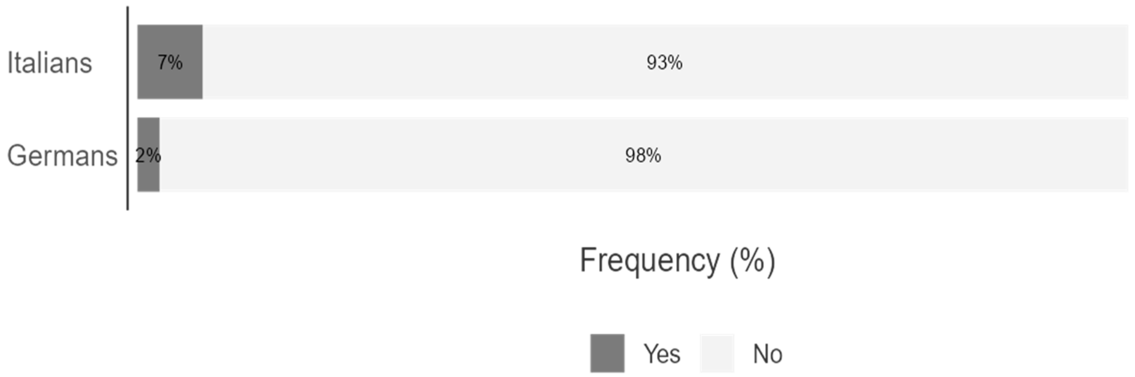


## 52_LOY2

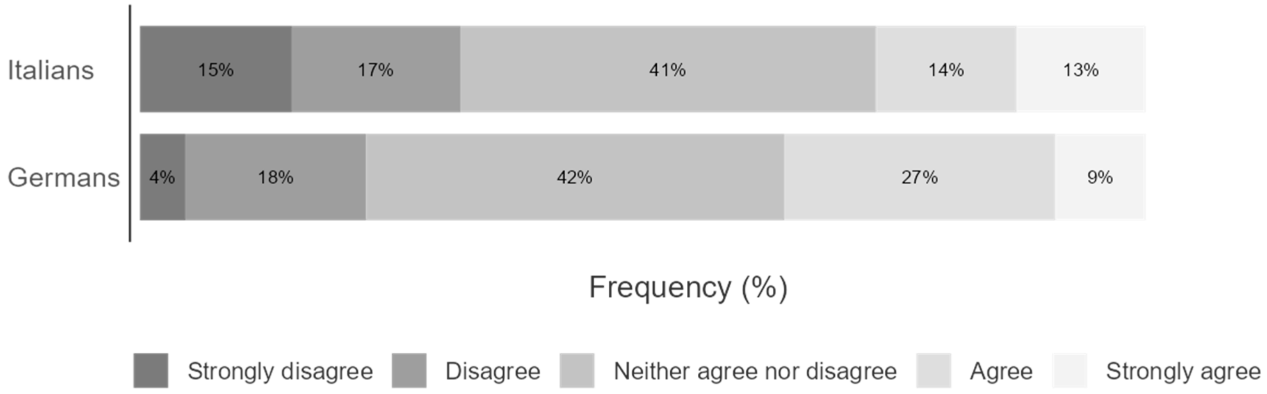


## 53_LOY3

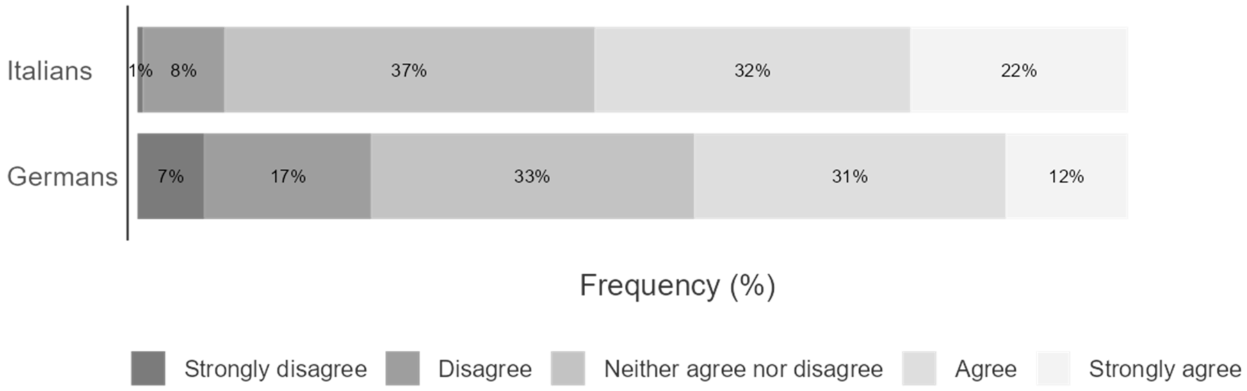

# EMOTIONAL APPEAL

10_EMA3

*Zoos have a good reputation*

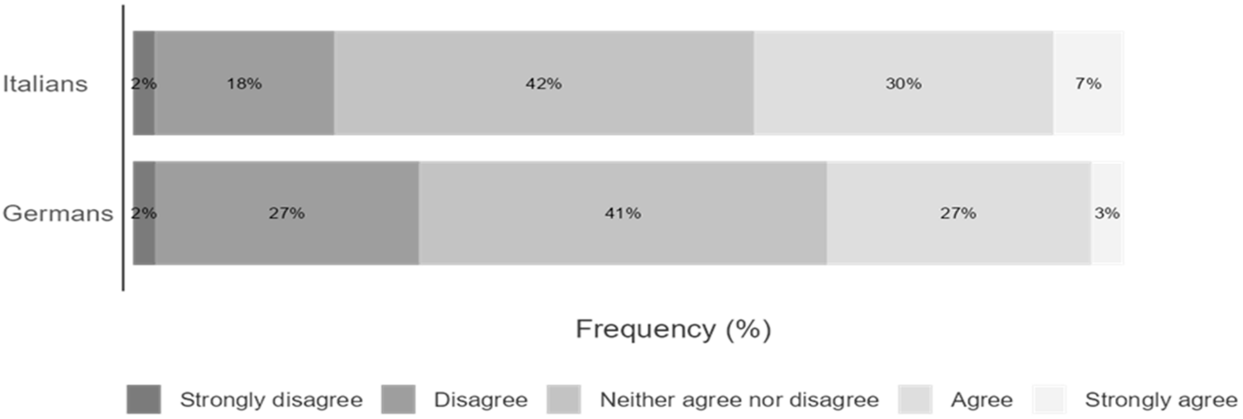


8_EMA1

*I trust in zoos.*

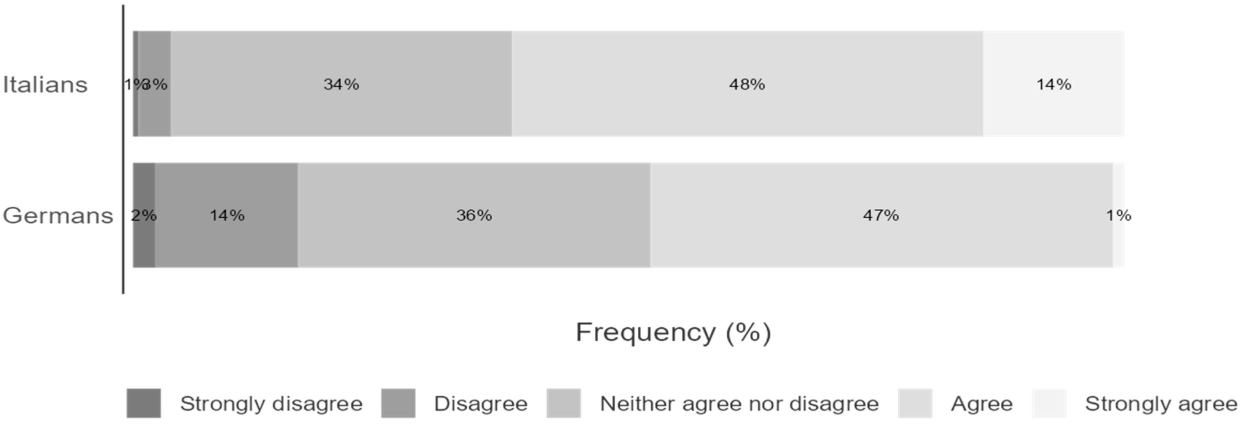


9_EMA2R

*I have negative feelings towards zoos.*

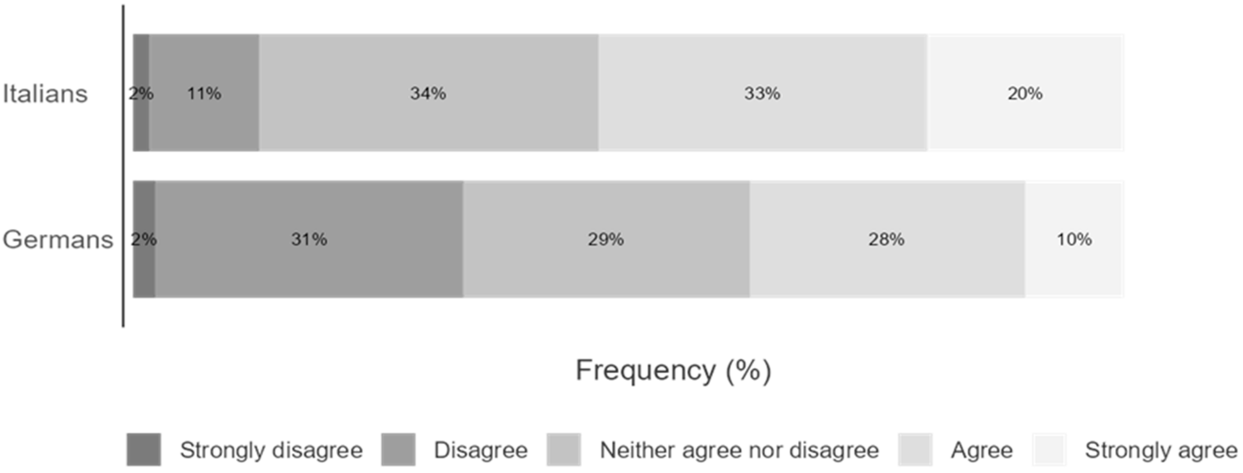

11_EMA4

*I admire and respect zoos.*

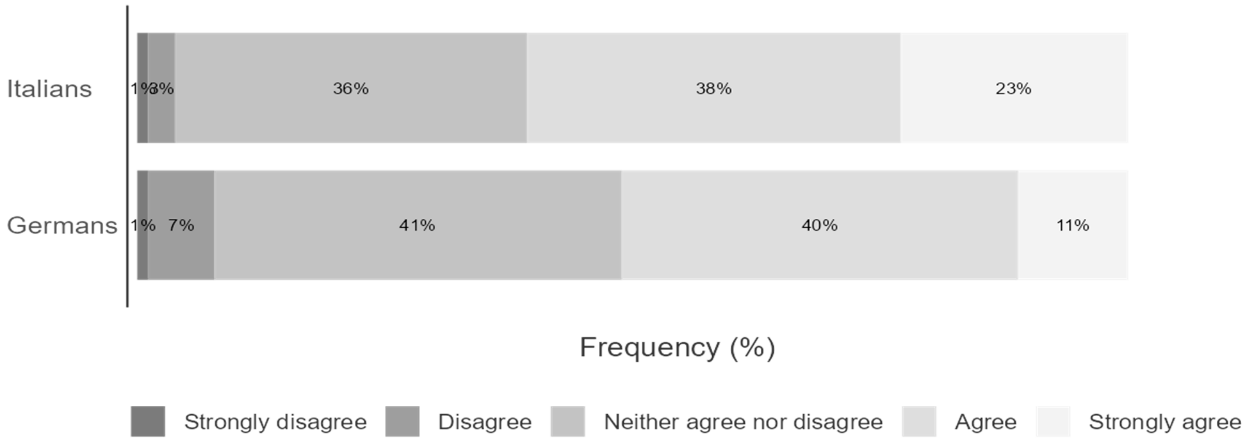


# KNOWLEDGE

22_KNOW1

*Are animals in zoos taken from the wild?*

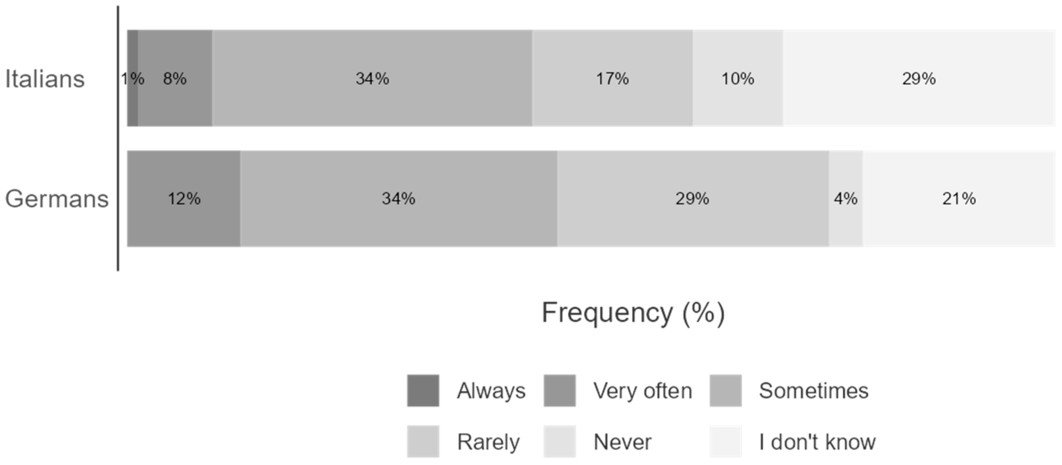

# Descriptives

| | Q2_0_NATION | Q2_1_FAM1 | Q2_2_FAM2 | Q2_3_FAM3 | Q2_4_FAM4 | Q2_5_FAM5 | Q2_6_FAM6 | Q2_8_EMA1 | Q2_9_EMA2R | Q2_10_EMA3 | Q2_11_EMA4 | 17_PERF1 | 18_PERF2 | 19_PERF3 | 21_PERF4 | 31_PERF5 | 20_PR_SR1 | 27_PR_SR2 |
|---|---|---|---|---|---|---|---|---|---|---|---|---|---|---|---|---|---|---|
| N | Italians | 178 | 175 | 169 | 164 | 159 | 172 | 183 | 181 | 182 | 183 | 184 | 184 | 183 | 183 | 182 | 184 | 184 |
| | Germans | 89 | 86 | 87 | 86 | 86 | 81 | 90 | 90 | 90 | 90 | 90 | 90 | 90 | 90 | 89 | 89 | 90 |
| Missing | Italians | 6 | 9 | 15 | 20 | 25 | 12 | 1 | 3 | 2 | 1 | 0 | 0 | 1 | 1 | 2 | 0 | 0 |
| | Germans | 1 | 4 | 3 | 4 | 4 | 9 | 0 | 0 | 0 | 0 | 0 | 0 | 0 | 0 | 1 | 1 | 0 |
| Mean | Italians | 2.13 | 1.70 | 1.93 | 1.21 | 1.48 | 2.72 | 3.72 | 3.59 | 3.22 | 3.79 | 3.58 | 3.70 | 3.57 | 3.87 | 3.40 | 3.88 | 3.83 |
| | Germans | 2.01 | 1.31 | 2.49 | 1.03 | 1.70 | 3.19 | 3.30 | 3.12 | 3.02 | 3.53 | 2.83 | 3.59 | 3.49 | 3.23 | 3.00 | 3.65 | 3.12 |
| Median | Italians | 2.00 | 2 | 2 | 1.00 | 1 | 3.00 | 4 | 4 | 3.00 | 4 | 4.00 | 4.00 | 4 | 4 | 3.00 | 4.00 | 4.00 |
| | Germans | 2 | 1.00 | 2 | 1.00 | 1.00 | 3 | 3.00 | 3.00 | 3.00 | 4.00 | 3.00 | 4.00 | 4.00 | 3.00 | 3 | 4 | 3.00 |
| Standard deviation | Italians | 0.883 | 0.737 | 1.05 | 0.467 | 0.778 | 0.934 | 0.768 | 0.983 | 0.902 | 0.867 | 0.896 | 0.901 | 0.854 | 0.812 | 0.865 | 0.900 | 0.861 |
| | Germans | 0.911 | 0.537 | 1.24 | 0.240 | 0.946 | 0.808 | 0.814 | 1.04 | 0.874 | 0.824 | 0.851 | 0.898 | 0.753 | 0.822 | 0.866 | 0.827 | 0.910 |
| Minimum | Italians | 1 | 1 | 1 | 1 | 1 | 1 | 1 | 1 | 1 | 1 | 1 | 1 | 1 | 1 | 1 | 1 | 1 |

# Descriptives

| | Q2_0_NATION | Q2_1_FAM1 | Q2_2_FAM2 | Q2_3_FAM3 | Q2_4_FAM4 | Q2_5_FAM5 | Q2_6_FAM6 | Q2_8_EMA1 | Q2_9_EMA2R | Q2_10_EMA3 | Q2_11_EMA4 | 17_PERF1 | 18_PERF2 | 19_PERF3 | 21_PERF4 | 31_PERF5 | 20_PR_SR1 | 27_PR_SR2 |
|---|---|---|---|---|---|---|---|---|---|---|---|---|---|---|---|---|---|---|
| | Germans | 1 | 1 | 1 | 1 | 1 | 1 | 1 | 1 | 1 | 1 | 1 | 1 | 1 | 1 | 1 | 2 | 1 |
| Maximum | Italians | 5 | 4 | 5 | 3 | 5 | 5 | 5 | 5 | 5 | 5 | 5 | 5 | 5 | 5 | 5 | 5 | 5 |
| | Germans | 5 | 3 | 5 | 3 | 5 | 5 | 5 | 5 | 5 | 5 | 4 | 5 | 5 | 5 | 5 | 5 | 5 |
| 25th percentile | Italians | 2.00 | 1.00 | 1.00 | 1.00 | 1.00 | 2.00 | 3.00 | 3.00 | 3.00 | 3.00 | 3.00 | 3.00 | 3.00 | 3.50 | 3.00 | 3.00 | 3.00 |
| | Germans | 1.00 | 1.00 | 2.00 | 1.00 | 1.00 | 3.00 | 3.00 | 2.00 | 2.00 | 3.00 | 2.00 | 3.00 | 3.00 | 3.00 | 3.00 | 3.00 | 3.00 |
| 50th percentile | Italians | 2.00 | 2.00 | 2.00 | 1.00 | 1.00 | 3.00 | 4.00 | 4.00 | 3.00 | 4.00 | 4.00 | 4.00 | 4.00 | 4.00 | 3.00 | 4.00 | 4.00 |
| | Germans | 2.00 | 1.00 | 2.00 | 1.00 | 1.00 | 3.00 | 3.00 | 3.00 | 3.00 | 4.00 | 3.00 | 4.00 | 4.00 | 3.00 | 3.00 | 4.00 | 3.00 |
| 75th percentile | Italians | 3.00 | 2.00 | 2.00 | 1.00 | 2.00 | 3.00 | 4.00 | 4.00 | 4.00 | 4.00 | 4.00 | 4.00 | 4.00 | 4.00 | 4.00 | 4.00 | 4.00 |
| | Germans | 2.00 | 2.00 | 3.00 | 1.00 | 2.00 | 4.00 | 4.00 | 4.00 | 4.00 | 4.00 | 3.00 | 4.00 | 4.00 | 4.00 | 4.00 | 4.00 | 4.00 |

**Q2_1_FAM1**

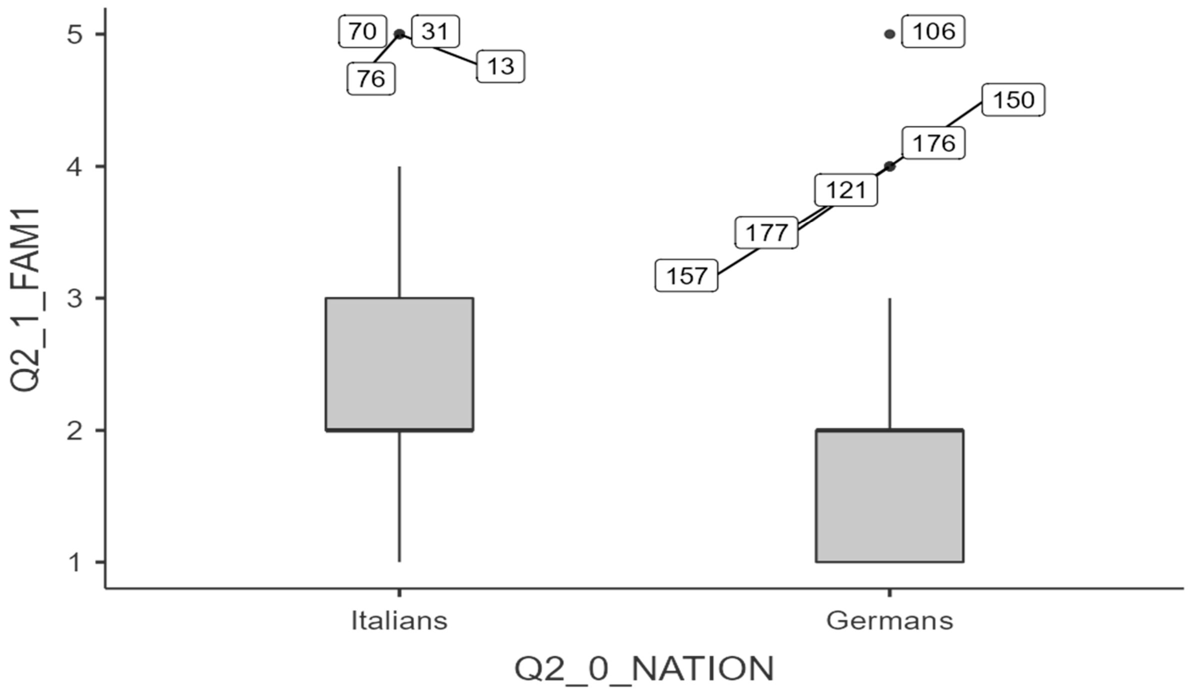


**Q2_2_FAM2**

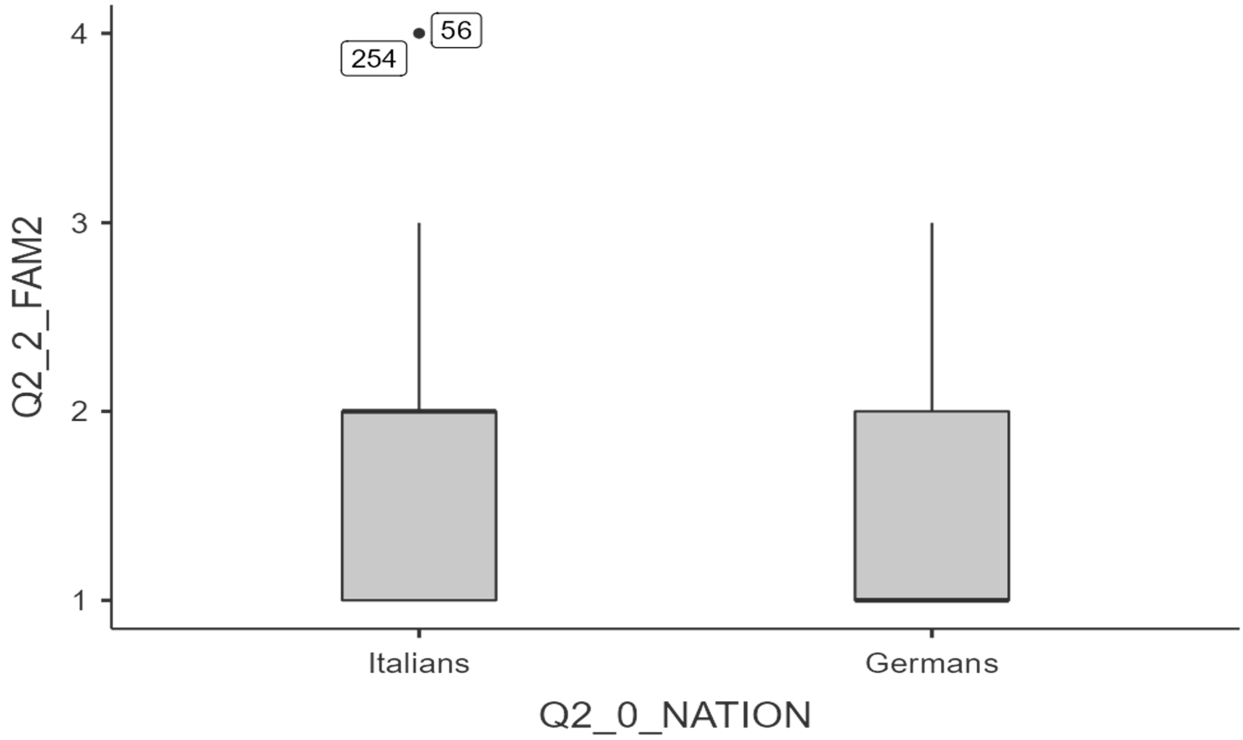

**Q2_3_FAM3**

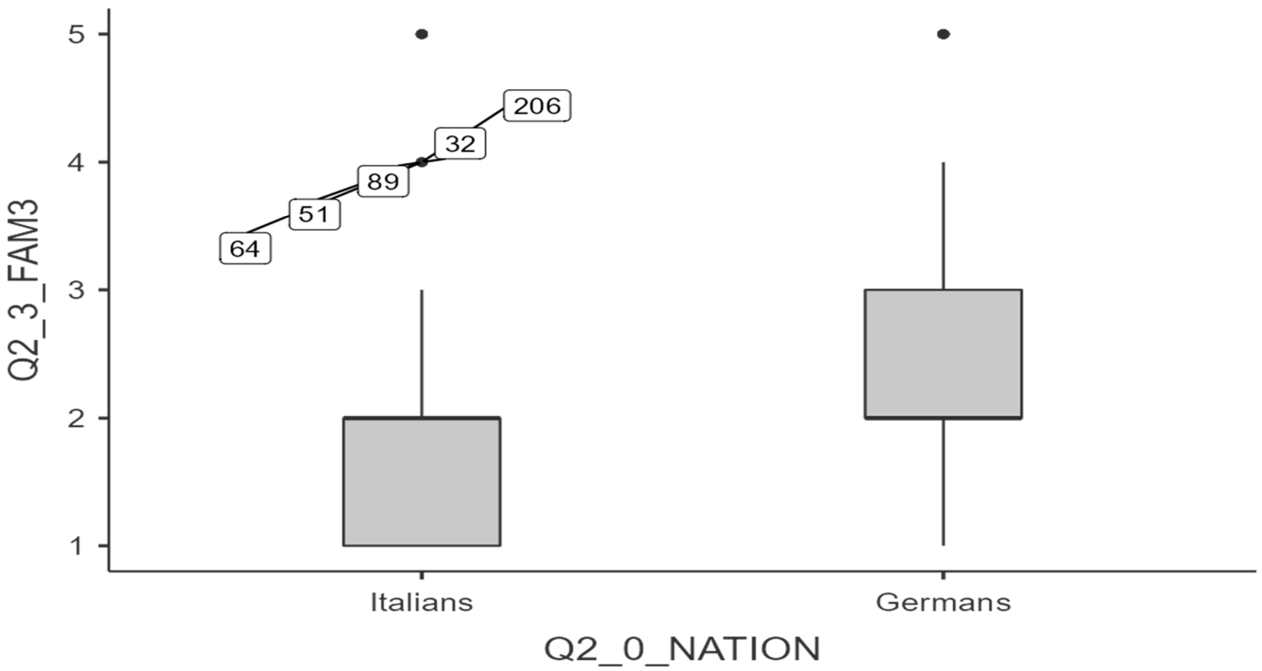


**Q2_4_FAM4**

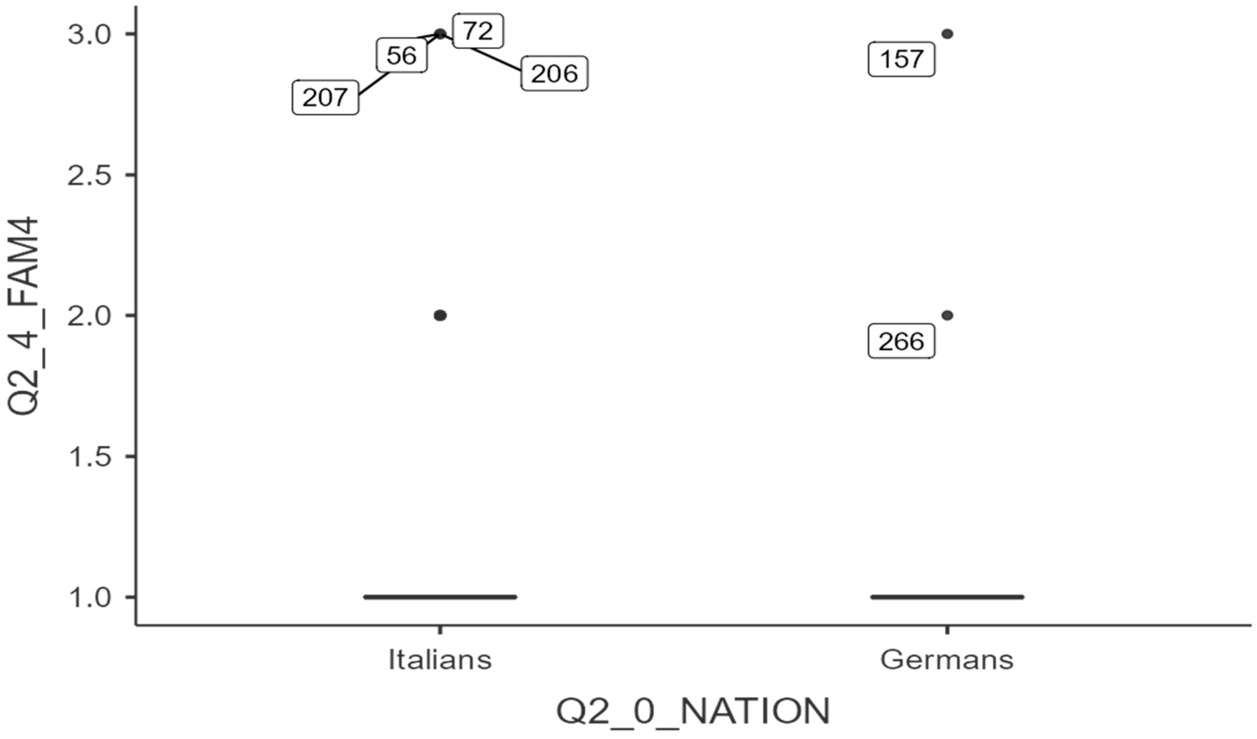

Q2_5_FAM5

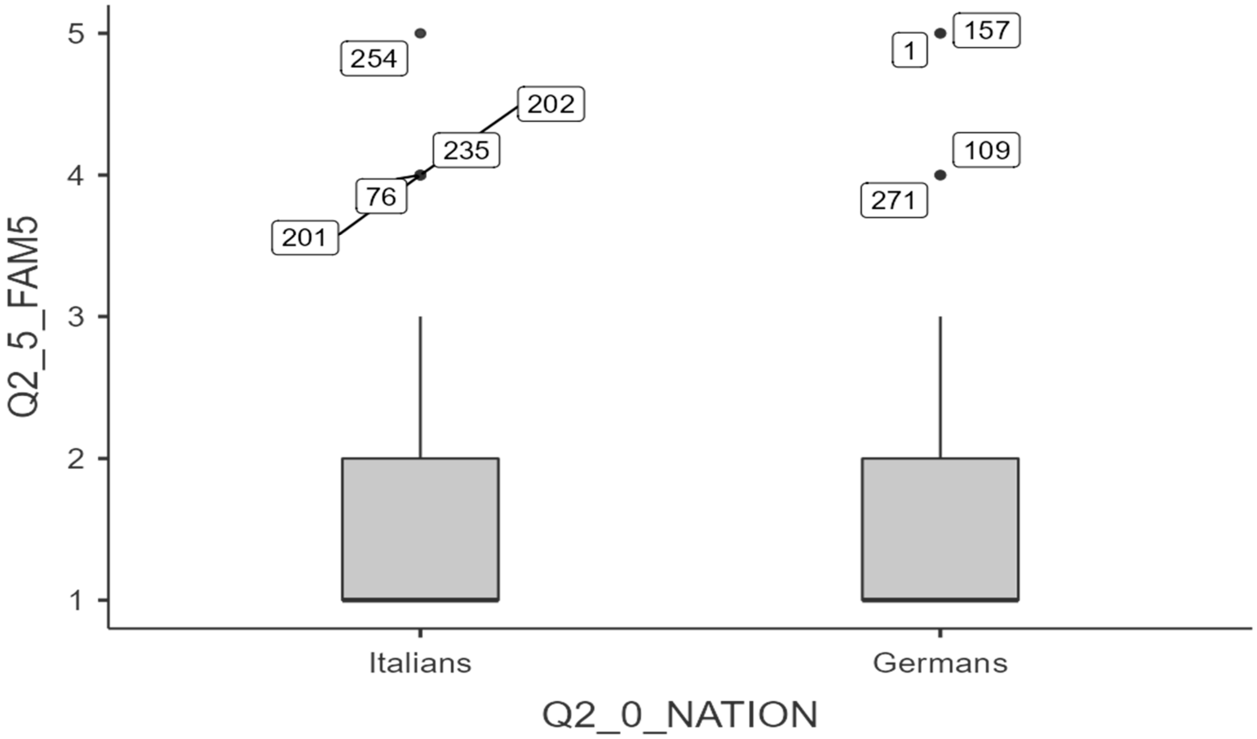


**Q2_6_FAM6**

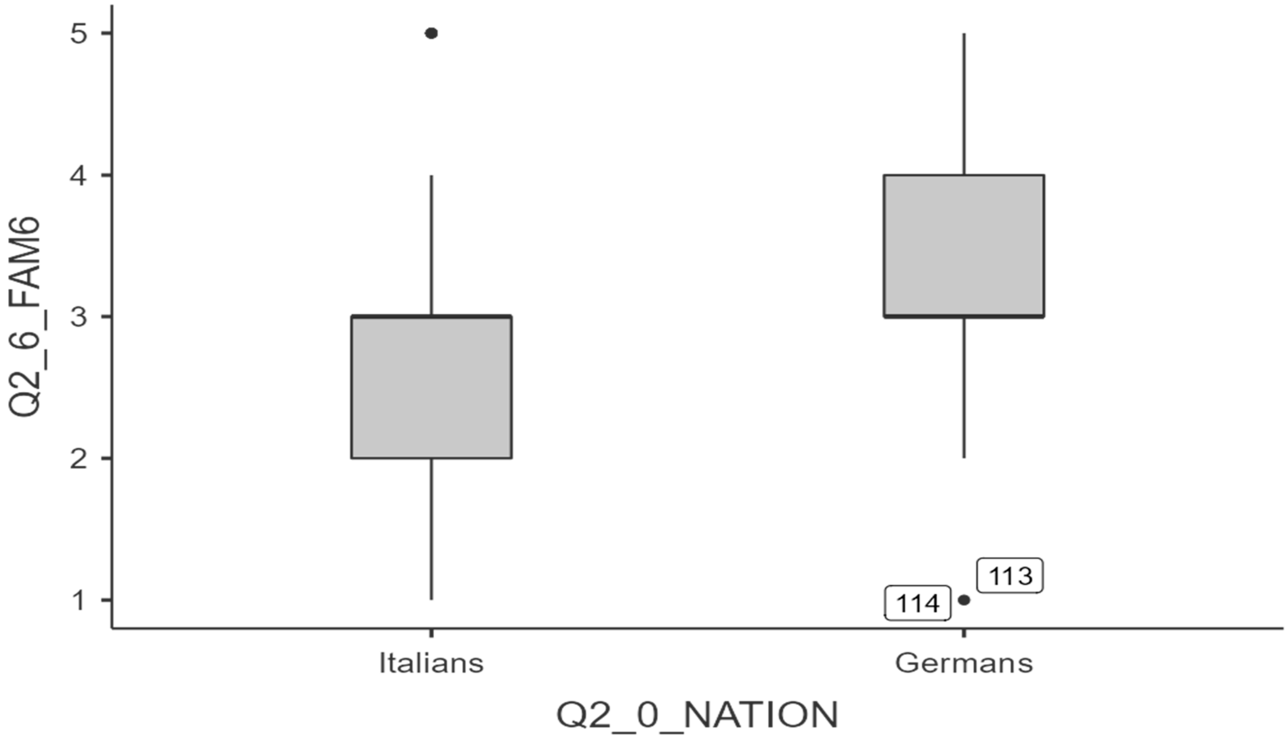

**Q2_8_EMA1**

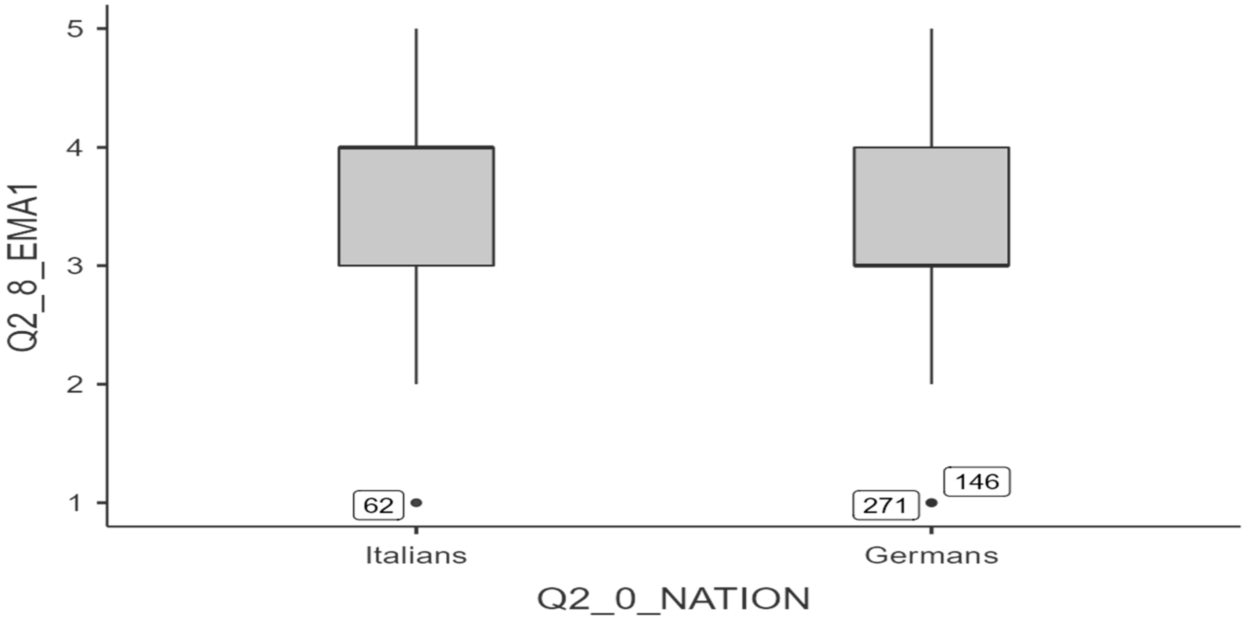


**Q2_9_EMA2R**

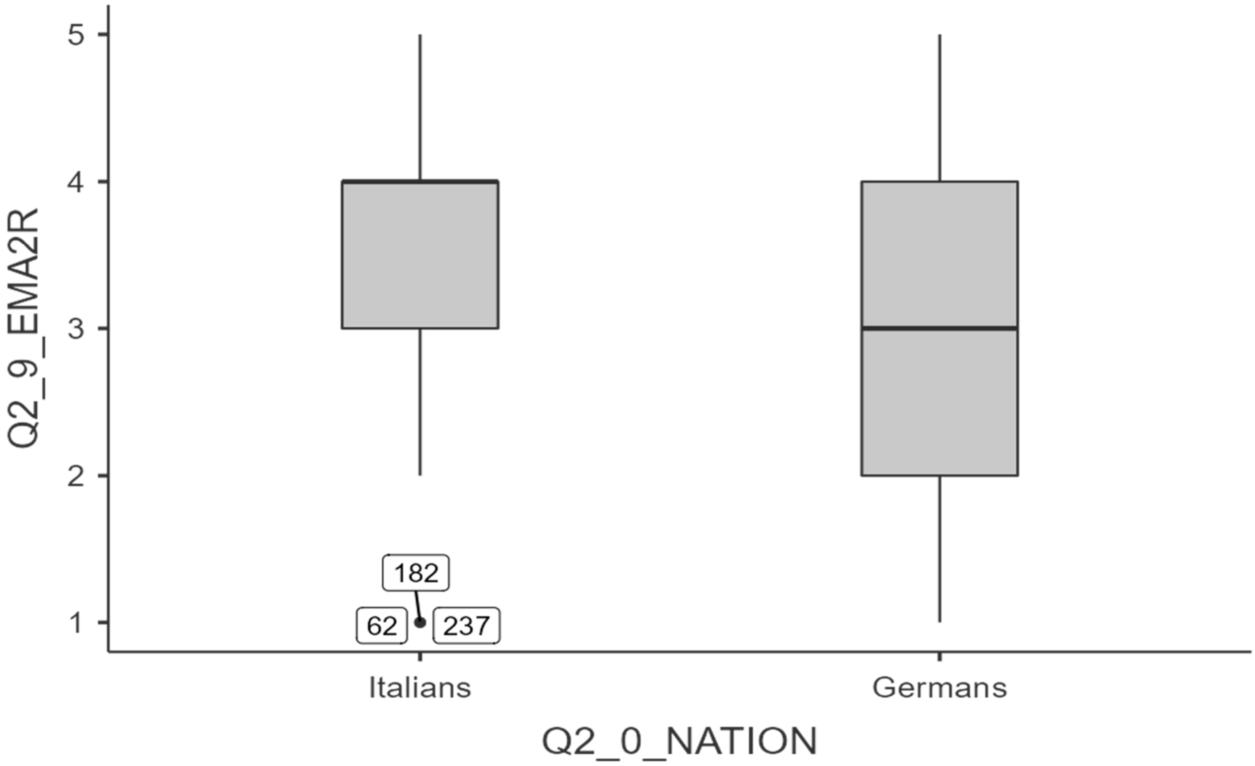

**Q2_10_EMA3**

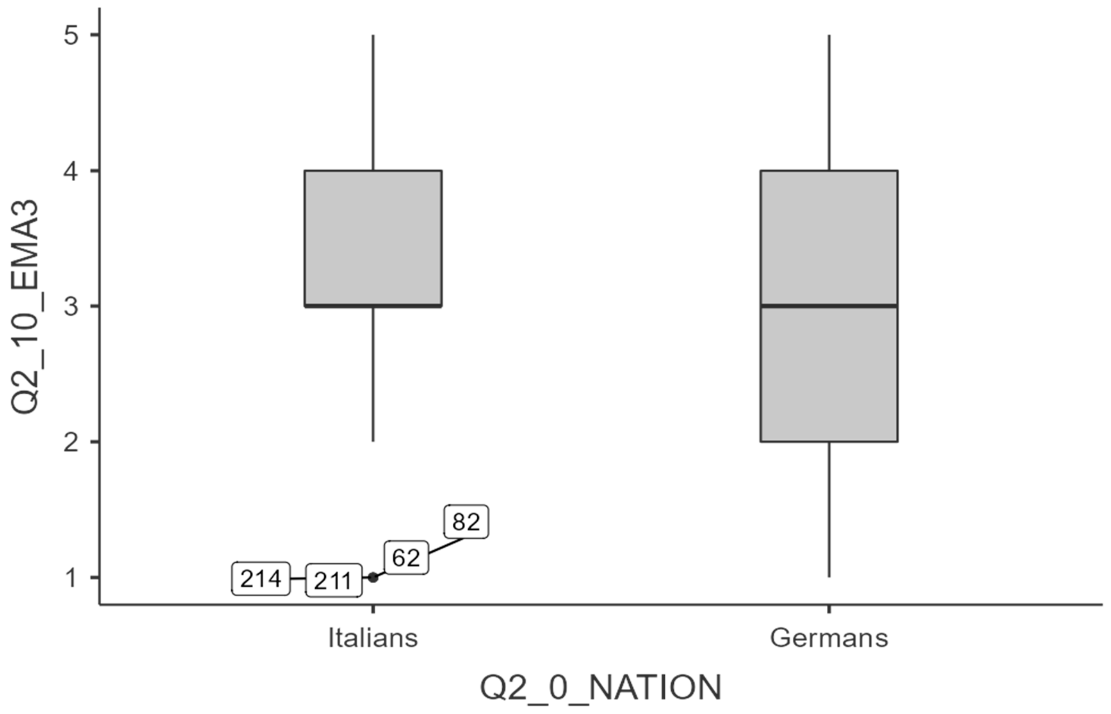


**Q2_11_EMA4**

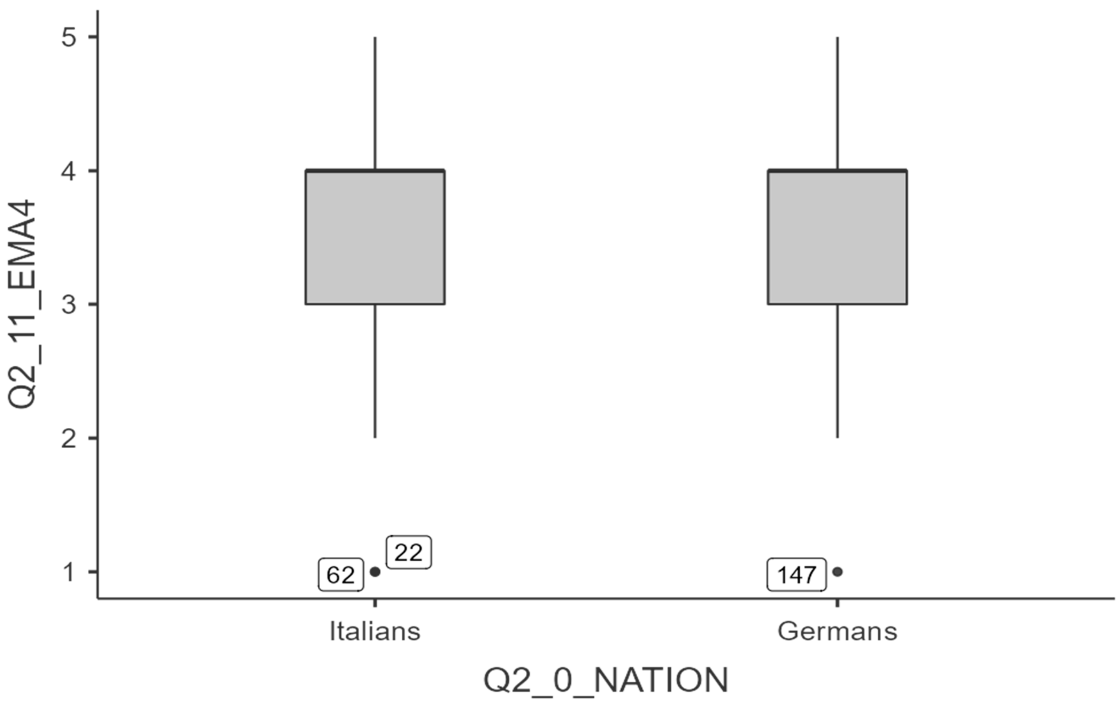

**17_PERF1**

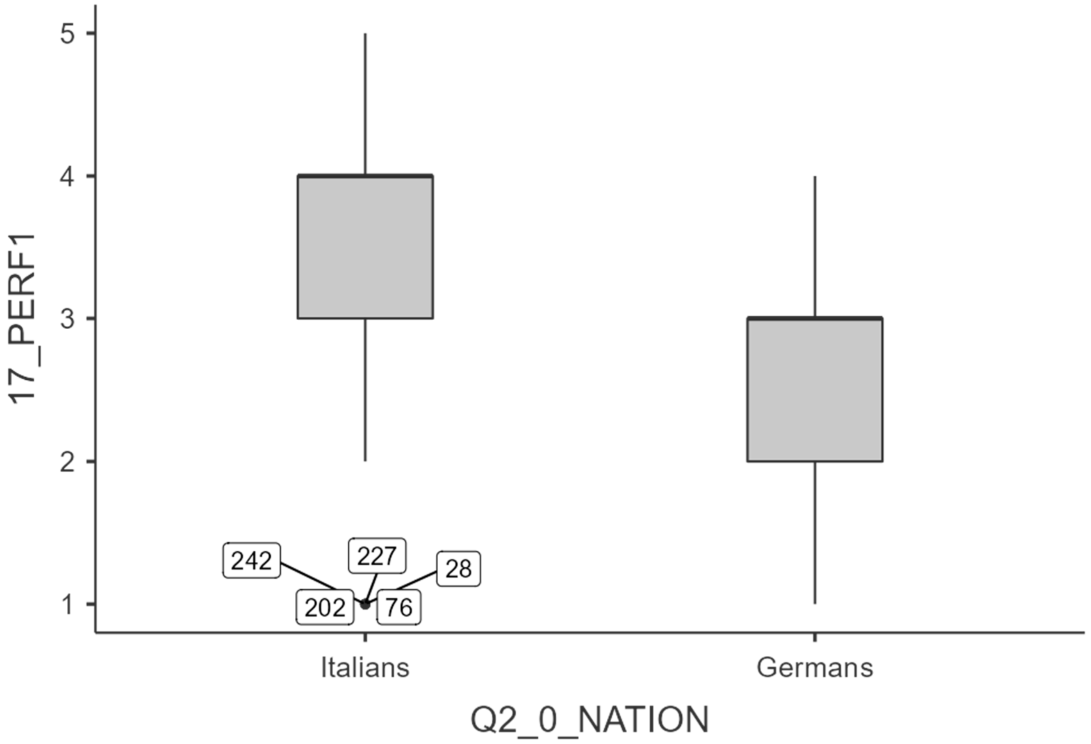


**18_PERF2**

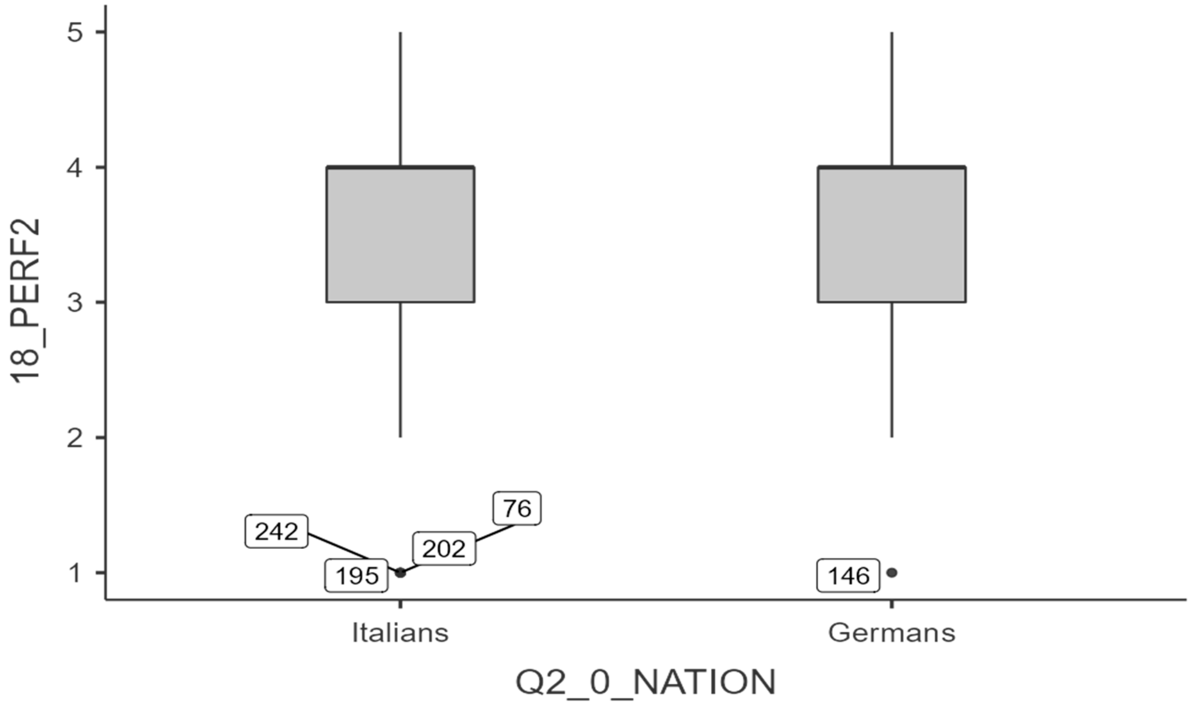

**19_PERF3**

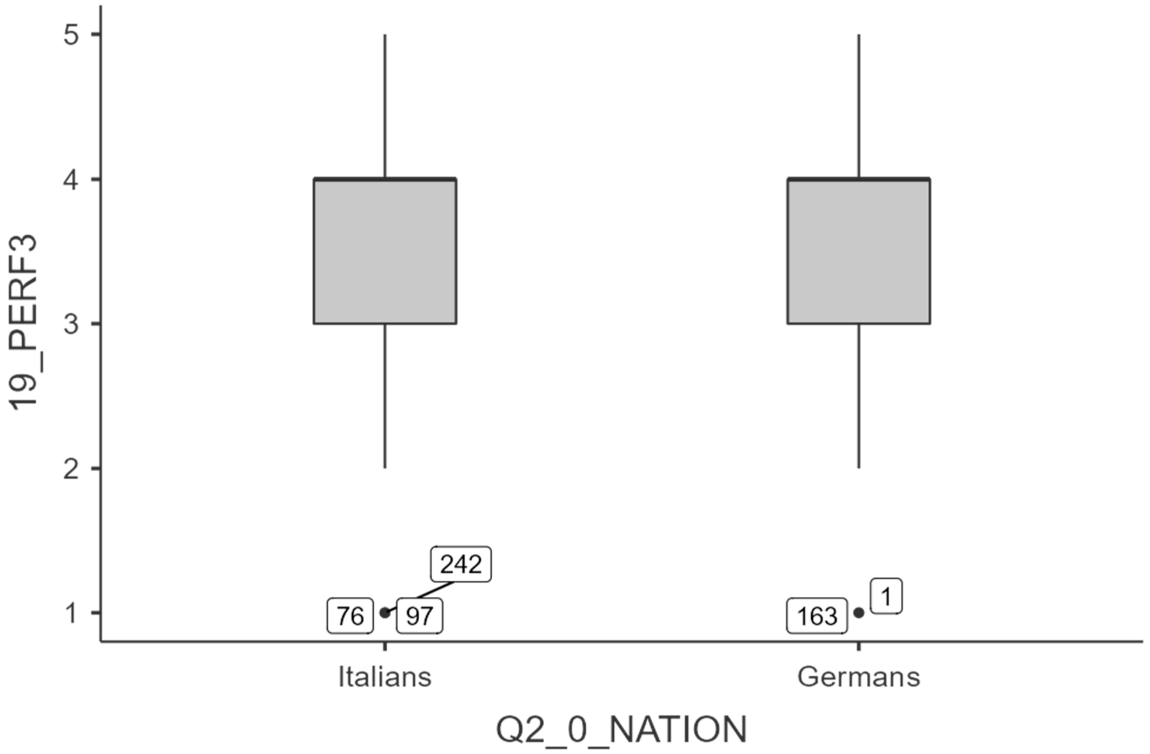


**21_PERF4**

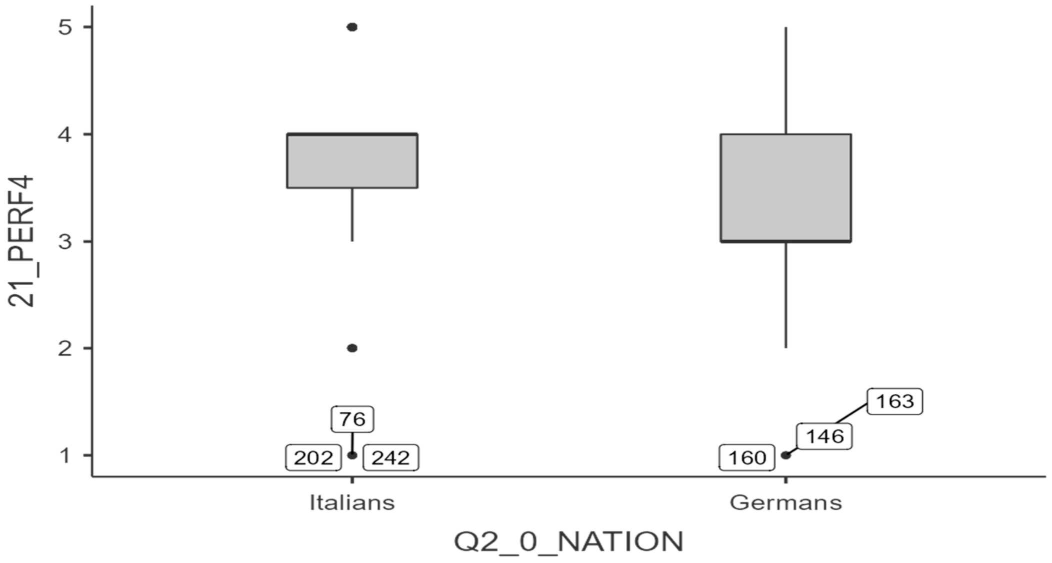

**31_PERF5**

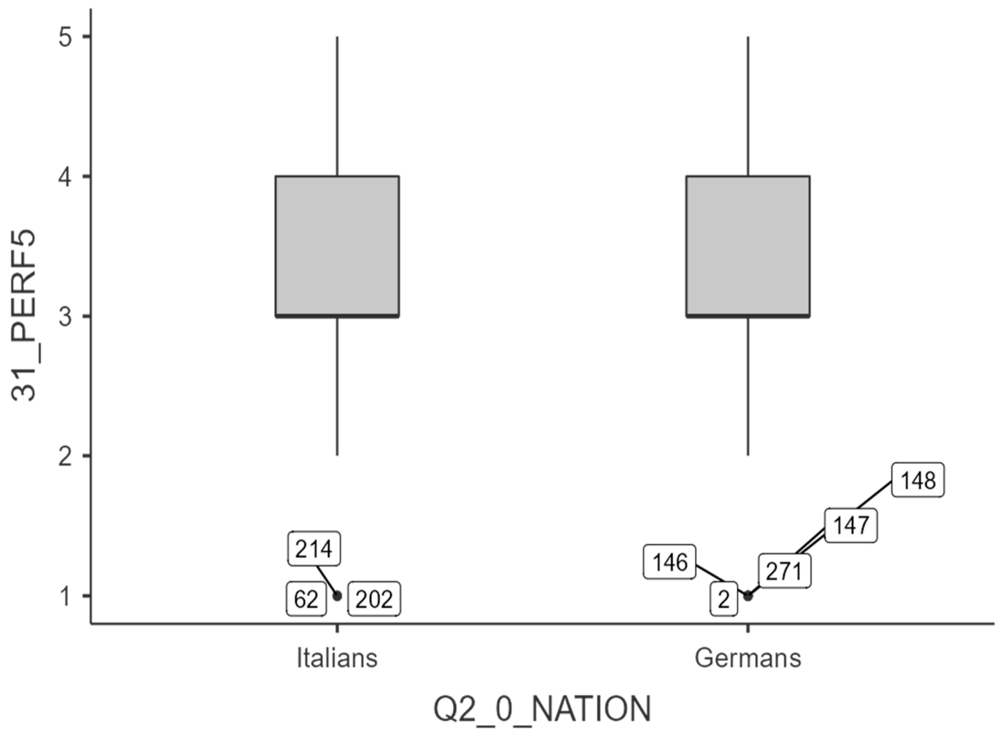


**20_PR_SR1**

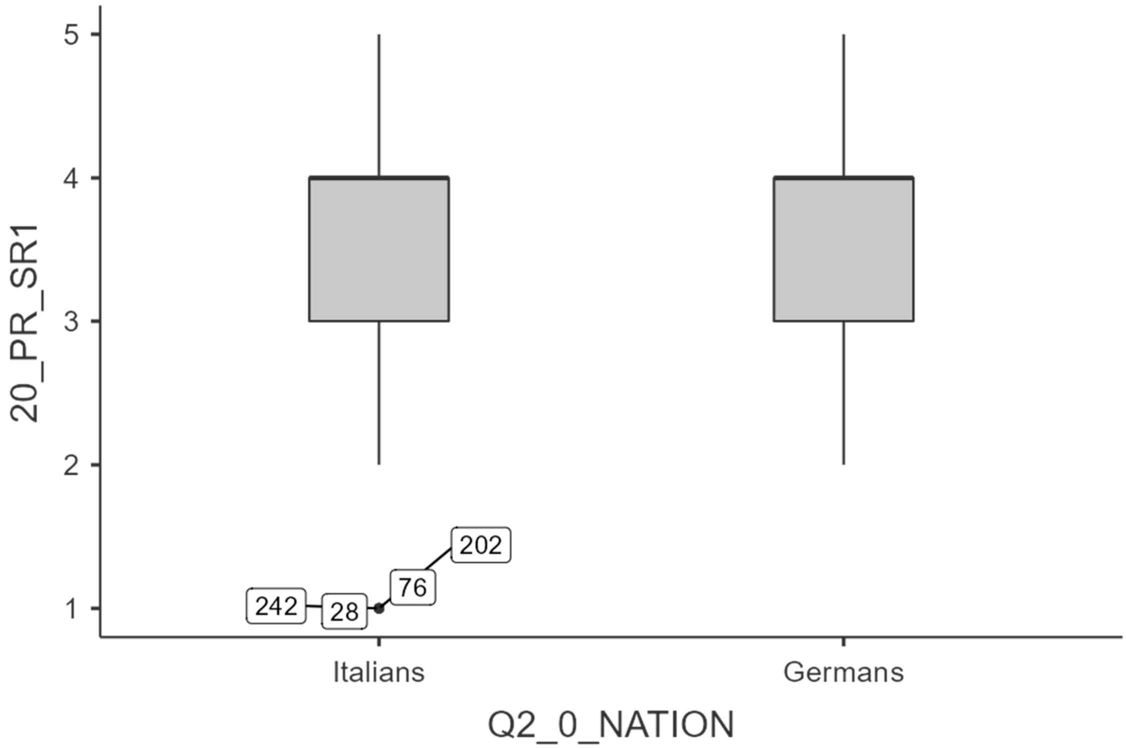

**PR_SR2**

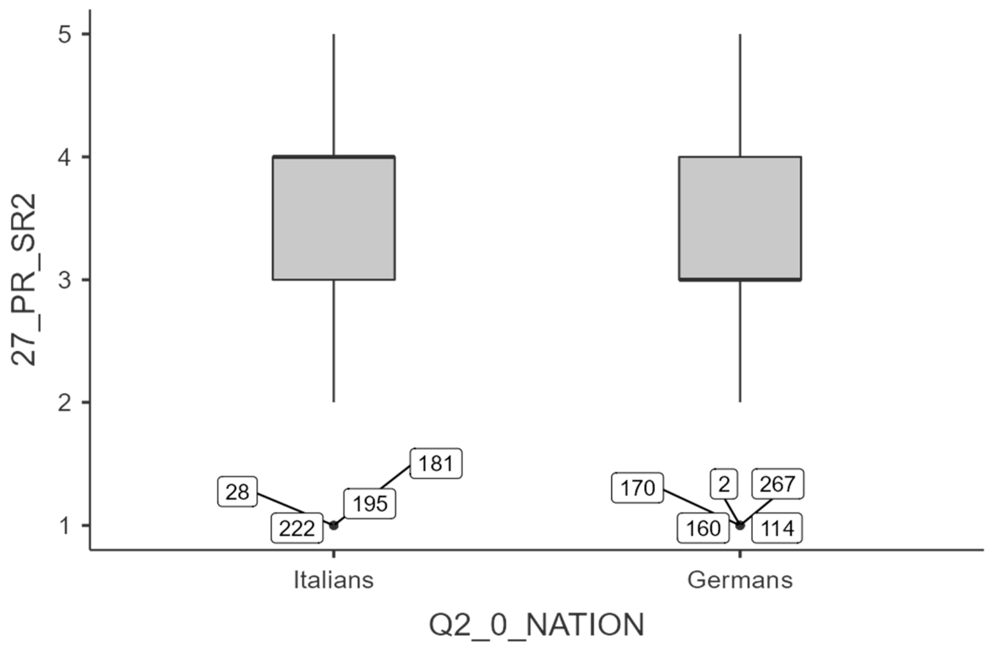

27_PR_SR2
5
4
3
2
1
28
222
195
181
170
2
267
160
114
Italians
Germans
Q2_0_NATION

Descriptives

| | Q2_0_NATION | 24_WORKP1 | 25_WORKP2 | 26_WORKP3 | 28_SOCRES1 | 29_SOCRES2 | 30_SOCRES3 | 34_VISION1R | 35_VISION2 | 36_VISION3 | 23_ETR1 | 33_ETR2 | 37_ETR3 | 38_ETR4 | 39_ETR5R | 32_ETR6 |
|---|---|---|---|---|---|---|---|---|---|---|---|---|---|---|---|---|
| N | Italians | 184 | 183 | 184 | 184 | 184 | 184 | 183 | 183 | 183 | 183 | 184 | 184 | 183 | 182 | 181 |
| | Germans | 90 | 90 | 90 | 89 | 89 | 87 | 90 | 90 | 90 | 90 | 90 | 90 | 90 | 90 | 89 |
| Missing | Italians | 0 | 1 | 0 | 0 | 0 | 0 | 1 | 1 | 1 | 1 | 0 | 0 | 1 | 2 | 3 |
| | Germans | 0 | 0 | 0 | 1 | 1 | 3 | 0 | 0 | 0 | 0 | 0 | 0 | 0 | 0 | 1 |
| Mean | Italians | 3.83 | 3.54 | 3.47 | 3.43 | 3.80 | 3.75 | 3.46 | 3.41 | 3.46 | 3.39 | 3.29 | 3.49 | 3.36 | 3.84 | 3.75 |
| | Germans | 3.62 | 3.32 | 3.26 | 3.22 | 3.21 | 3.21 | 3.38 | 3.12 | 3.13 | 2.99 | 2.90 | 3.26 | 3.02 | 3.54 | 3.29 |
| Median | Italians | 4.00 | 4 | 3.00 | 3.00 | 4.00 | 4.00 | 3 | 3 | 3 | 3 | 3.00 | 3.50 | 3 | 4.00 | 4 |
| | Germans | 4.00 | 3.00 | 3.00 | 3 | 3 | 3 | 3.00 | 3.00 | 3.00 | 3.00 | 3.00 | 3.00 | 3.00 | 3.00 | 3 |
| Standard deviation | Italians | 0.795 | 0.768 | 0.753 | 0.721 | 0.665 | 0.703 | 0.709 | 0.735 | 0.768 | 0.709 | 0.774 | 0.724 | 0.664 | 0.883 | 0.804 |
| | Germans | 0.787 | 0.668 | 0.758 | 0.653 | 0.790 | 0.701 | 0.758 | 0.557 | 0.767 | 0.757 | 0.765 | 0.696 | 0.474 | 0.752 | 0.815 |
| Minimum | Italians | 1 | 1 | 1 | 1 | 1 | 1 | 2 | 1 | 2 | 2 | 1 | 2 | 2 | 1 | 1 |
| | Germans | 1 | 1 | 1 | 1 | 1 | 1 | 1 | 1 | 2 | 1 | 1 | 1 | 2 | 2 | 1 |
| Maximum | Italians | 5 | 5 | 5 | 5 | 5 | 5 | 5 | 5 | 5 | 5 | 5 | 5 | 5 | 5 | 5 |

Descriptives

| | Q2_0_NATION | 24_WORKP1 | 25_WORKP2 | 26_WORKP3 | 28_SOCRES1 | 29_SOCRES2 | 30_SOCRES3 | 34_VISION1R | 35_VISION2 | 36_VISION3 | 23_ETR1 | 33_ETR2 | 37_ETR3 | 38_ETR4 | 39_ETR5R | 32_ETR6 |
|---|---|---|---|---|---|---|---|---|---|---|---|---|---|---|---|---|
| | Germans | 5 | 4 | 5 | 5 | 5 | 5 | 5 | 4 | 5 | 5 | 4 | 5 | 4 | 5 | 5 |
| 25th percentile | Italians | 3.00 | 3.00 | 3.00 | 3.00 | 3.75 | 3.00 | 3.00 | 3.00 | 3.00 | 3.00 | 3.00 | 3.00 | 3.00 | 3.00 | 3.00 |
| | Germans | 3.00 | 3.00 | 3.00 | 3.00 | 3.00 | 3.00 | 3.00 | 3.00 | 3.00 | 3.00 | 2.00 | 3.00 | 3.00 | 3.00 | 3.00 |
| 50th percentile | Italians | 4.00 | 4.00 | 3.00 | 3.00 | 4.00 | 4.00 | 3.00 | 3.00 | 3.00 | 3.00 | 3.00 | 3.50 | 3.00 | 4.00 | 4.00 |
| | Germans | 4.00 | 3.00 | 3.00 | 3.00 | 3.00 | 3.00 | 3.00 | 3.00 | 3.00 | 3.00 | 3.00 | 3.00 | 3.00 | 3.00 | 3.00 |
| 75th percentile | Italians | 4.00 | 4.00 | 4.00 | 4.00 | 4.00 | 4.00 | 4.00 | 4.00 | 4.00 | 4.00 | 4.00 | 4.00 | 4.00 | 4.00 | 4.00 |
| | Germans | 4.00 | 4.00 | 4.00 | 4.00 | 4.00 | 4.00 | 4.00 | 3.00 | 4.00 | 3.00 | 3.00 | 4.00 | 3.00 | 4.00 | 4.00 |

Descriptives

| | Q2_0_NATION | 50_PWM1 | 51_PWM2 | 7_LOY1 | 52_LOY2 | 53_LOY3 |
|---|---|---|---|---|---|---|
| N | Italians | 184 | 184 | 182 | 179 | 182 |
| | Germans | 90 | 89 | 90 | 89 | 89 |
| Missing | Italians | 0 | 0 | 2 | 5 | 2 |
| | Germans | 0 | 1 | 0 | 1 | 1 |
| Mean | Italians | 4.20 | 4.09 | 1.93 | 2.93 | 3.66 |
| | Germans | 3.37 | 3.34 | 1.98 | 3.18 | 3.26 |
| Median | Italians | 4.00 | 4.00 | 2.00 | 3 | 4.00 |
| | Germans | 3.00 | 3 | 2.00 | 3 | 3 |
| Standard deviation | Italians | 0.908 | 0.883 | 0.249 | 1.19 | 0.930 |
| | Germans | 1.28 | 1.13 | 0.148 | 0.984 | 1.09 |
| Minimum | Italians | 1 | 1 | 1 | 1 | 1 |
| | Germans | 1 | 1 | 1 | 1 | 1 |
| Maximum | Italians | 5 | 5 | 2 | 5 | 5 |
| | Germans | 5 | 5 | 2 | 5 | 5 |
| 25th percentile | Italians | 3.00 | 3.75 | 2.00 | 2.00 | 3.00 |
| | Germans | 3.00 | 3.00 | 2.00 | 3.00 | 3.00 |
| 50th percentile | Italians | 4.00 | 4.00 | 2.00 | 3.00 | 4.00 |
| | Germans | 3.00 | 3.00 | 2.00 | 3.00 | 3.00 |
| 75th percentile | Italians | 5.00 | 5.00 | 2.00 | 4.00 | 4.00 |
| | Germans | 4.00 | 4.00 | 2.00 | 4.00 | 4.00 |

**50_PWM1**

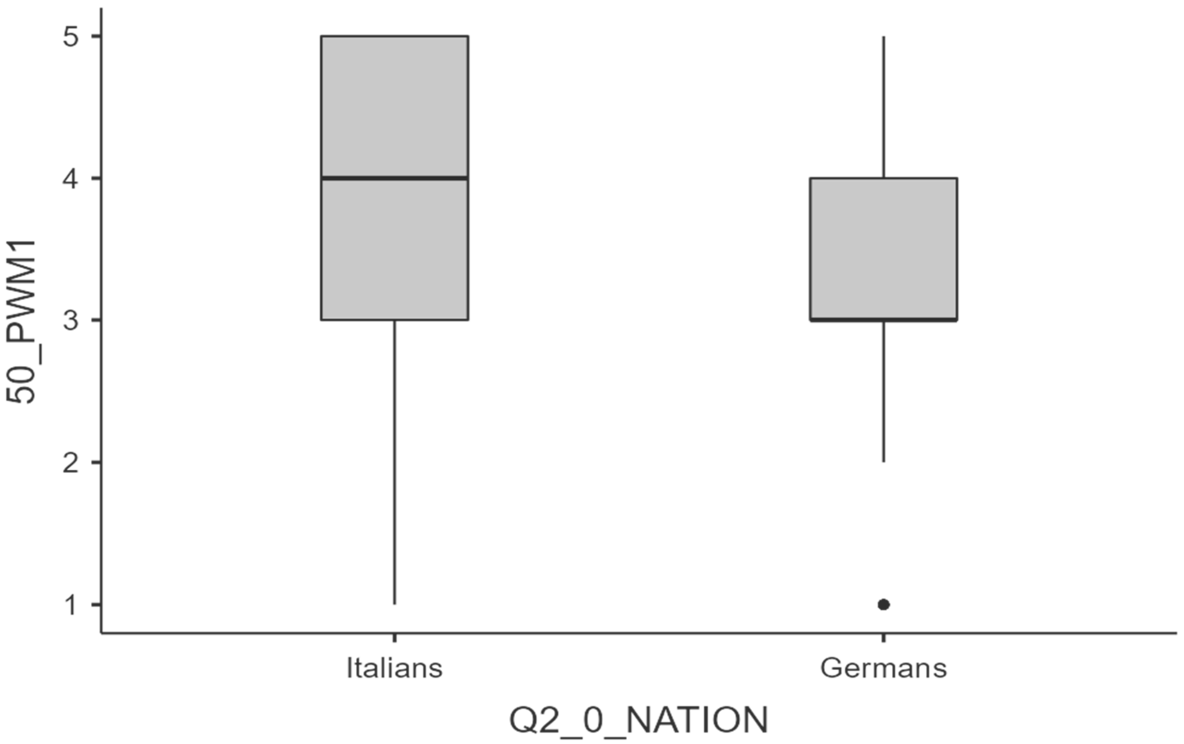


**51_PWM2**

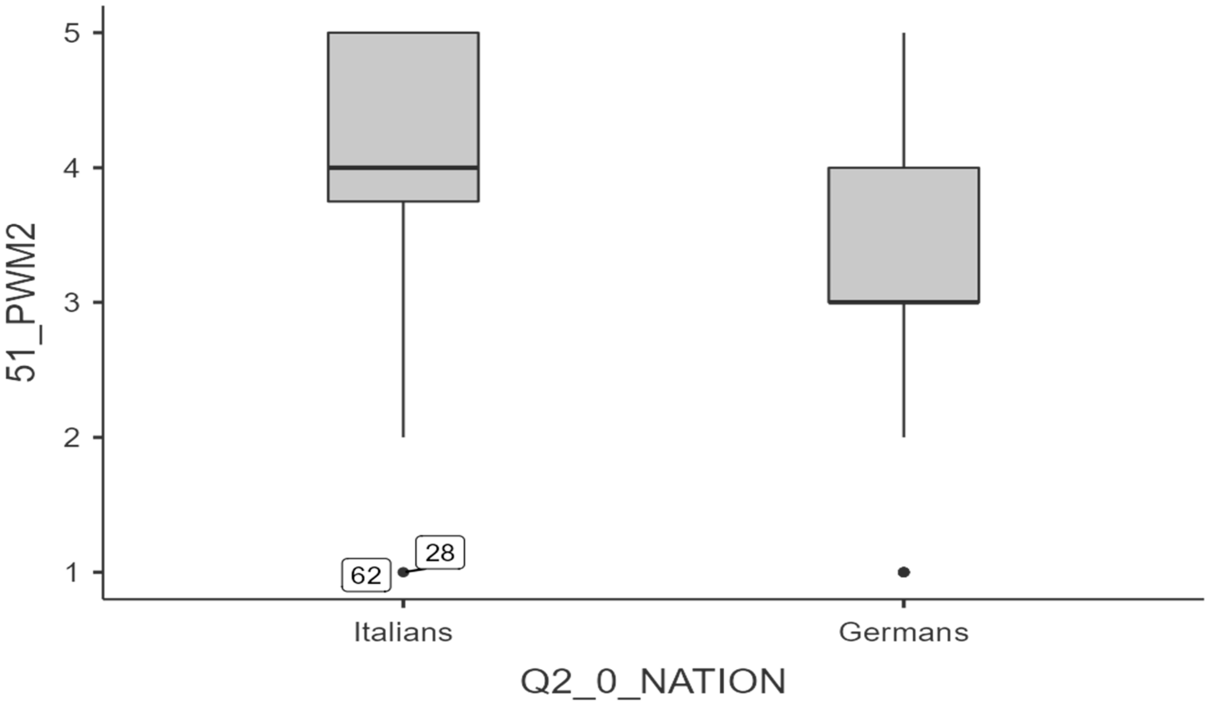

**7_LOY1**

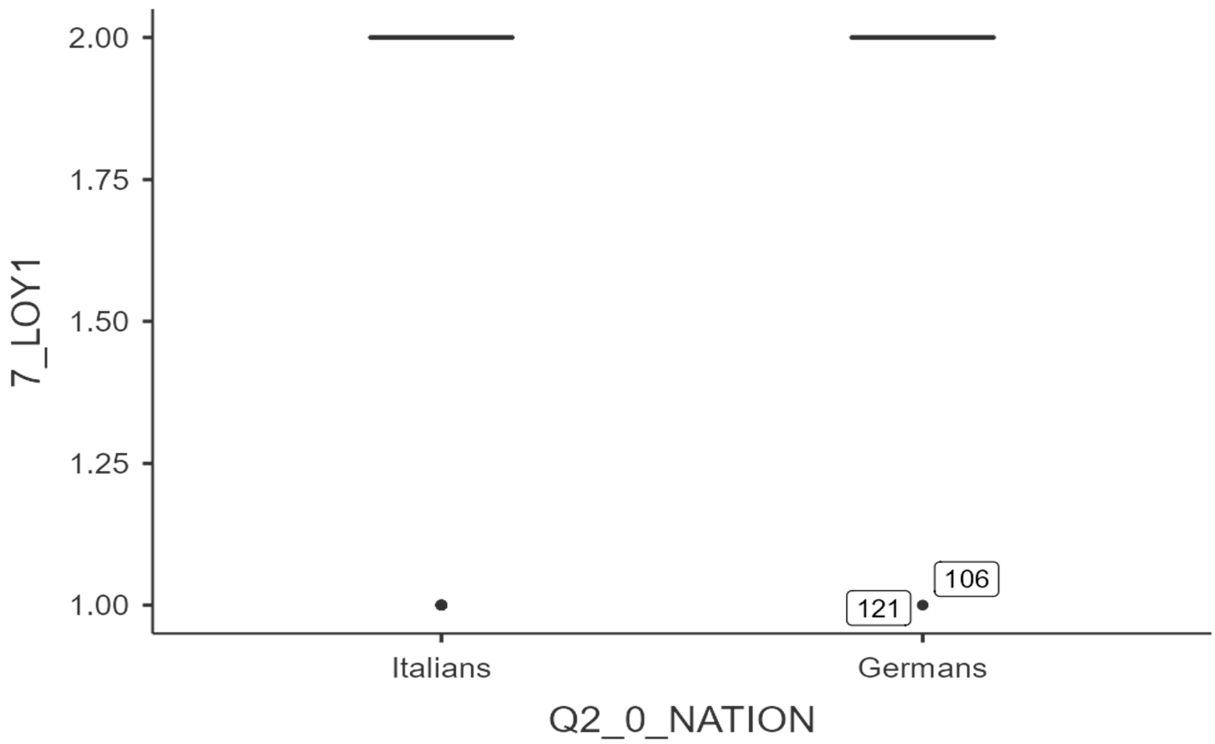
2.00
1.75
1.50
1.25
1.00
7_LOY1
106
121
Italians
Germans
Q2_0_NATION

**2_LOY2**

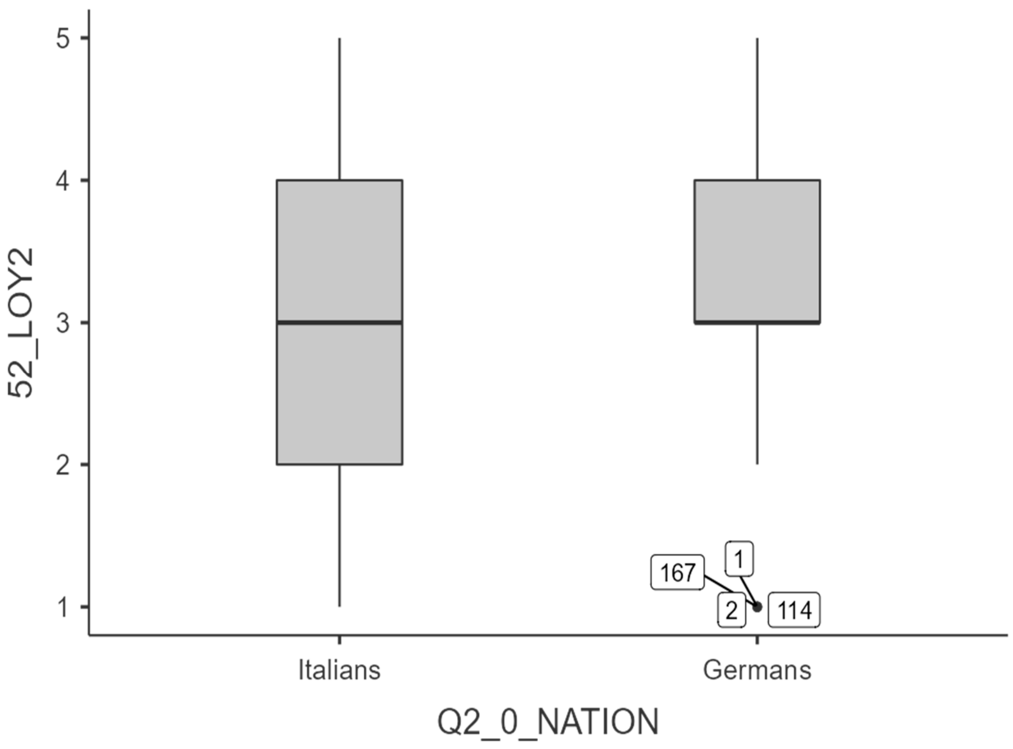
5
4
3
2
1
52_LOY2
167
1
2
114
Italians
Germans
Q2_0_NATION

**53_LOY3**

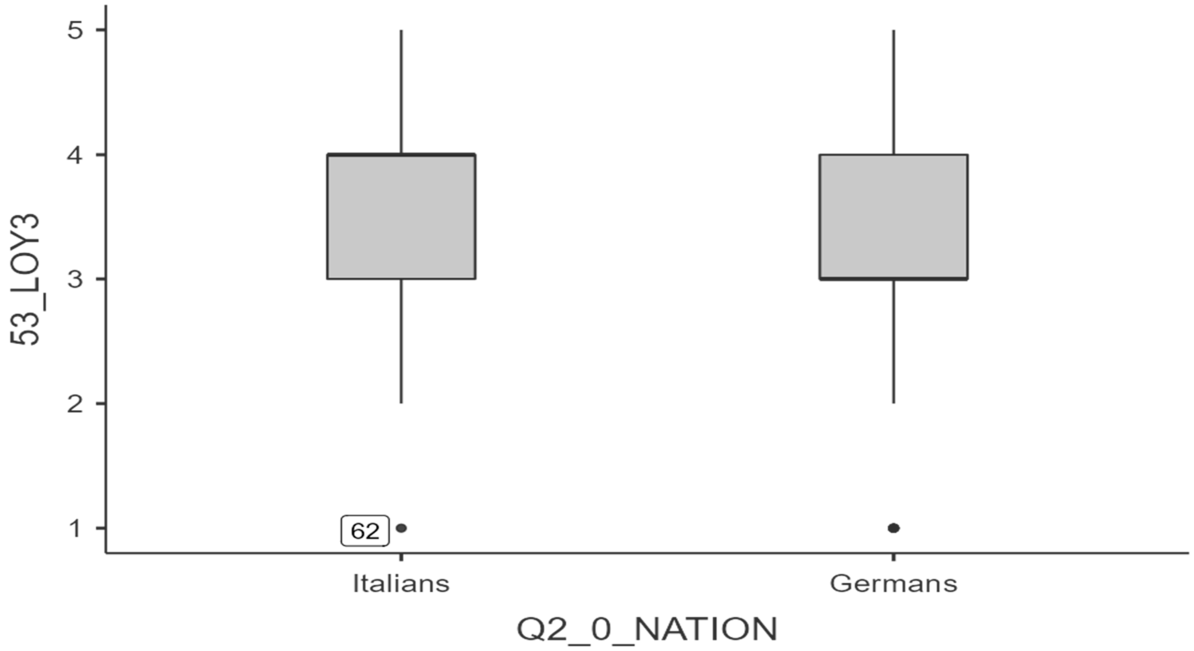

Supplement: Supplementary file 1 [file animals-12-02802-s001.zip › S2.pdf]
